# Patient-Specific Subperiosteal Implants for Oral and Maxillofacial Rehabilitation: A Scoping Review Across Indications, from Established Full-Arch Use to Emerging Single-Tooth and Oncologic Applications

**DOI:** 10.3390/jcm15135220

**Published:** 2026-07-03

**Authors:** Luigi Angelo Vaira, Hareem Qadeer, Andrea Biglio, Sebastiano Stellino, Jerome R. Lechien, Antonino Maniaci, Fabio Maglitto, Giuseppe Consorti, Giulio Cirignaco, Carlos Navarro-Cuéllar, Giovanni Salzano, Valentino Vellone, Marco Roy, Javier Herce-López, Marshall M. Freilich, Álvaro Tofé-Povedano, Casper van den Borre, Maurice Y. Mommaerts, Giacomo De Riu

**Affiliations:** 1 Maxillofacial Surgery Operative Unit, Department of Medicine, Surgery and Pharmacy, University of Sassari, 07100 Sassari, Italy; h.qadeerahmad@studenti.uniss.it (H.Q.); andreabiglio@gmail.com (A.B.); sebastianostellino@hotmail.it (S.S.); gderiu@uniss.it (G.D.R.); 2 PhD School of Biomedical Sciences, Department of Biomedical Science, University of Sassari, 07100 Sassari, Italy; 3 Department of Surgery, Mons School of Medicine, Research Institute for Health Sciences and Technology, University of Mons (UMons), 7000 Mons, Belgium; jerome.lechien@umons.ac.be; 4 Department of Otolaryngology-Head Neck Surgery, Elsan Polyclinic of Poitiers, 86000 Poitiers, France; 5 Department of Medicine and Surgery, University of Enna Kore, 94100 Enna, Italy; antonino.maniaci@unikore.it; 6 Head and Neck Section, Department of Neurosciences, Reproductive and Odontostomatological Science, Federico II University of Naples, 80138 Naples, Italy; fabio.maglitto@unina.it (F.M.); giovanni.salzano@unina.it (G.S.); 7 Division of Maxillofacial Surgery, Department of Neurological Sciences, Marche University Hospitals—Umberto I, 60121 Ancona, Italy; giuseppe.consorti@ospedaliriuniti.marche.it (G.C.); giuliocirignaco@gmail.com (G.C.); 8 Department of Biomedical Sciences and Public Health, Polytechnic University of Marche, 60121 Ancona, Italy; 9 Department of Medicine, Section of Maxillo-Facial Surgery, University of Siena, Viale Bracci, 53100 Siena, Italy; 10 Maxillofacial Surgery Department, Hospital Gregorio Marañon, Universidad Complutense de Madrid, 28007 Madrid, Spain; canava03@ucm.es; 11 Department of Life Science, Health, and Health Professions, Università degli Studi “Link”, 00165 Rome, Italy; v.vellone@unilink.it; 12 Department of Prosthodontics and Gerostomatology, Poznan University of Medical Sciences, Ul Bukowska 70, 60-792 Poznan, Poland; marcoroy@ump.edu.pl; 13 Department of Oral and Maxillofacial Surgery, Universidad CEU Fernando III, 41930 Bormujos, Spain; javier.hercelopez@ceu.es; 14 Division of Oral and Maxillofacial Surgery, Humber River Hospital, Toronto, ON M3M 0B2, Canada; drfreilich@rogers.com; 15 Department of Oral and Maxillofacial Surgery, Puerta del Mar University Hospital, 11009 Cádiz, Spain; alvarotofe@gmail.com; 16 Department of Oral and Maxillofacial Surgery, AZ Sint-Elisabeth Hospital, 9620 Zottegem, Belgium; casper.van.den.borre@vub.be; 17 Department of Oral and Maxillofacial Surgery, AZ Glorieux Hospital, 9600 Ronse, Belgium; 18 Face Ahead Surgicenter—Private Clinic—AMSJI Center of Excellence, 2018 Antwerpen, Belgium; mauricemommaerts@cadskills.eu; 19 Faculty of Medicine and Pharmacy, VUB Health Campus, Vrije Universiteit Brussel, 1090 Brussels, Belgium

**Keywords:** subperiosteal implant, patient-specific implant, custom-made implant, CAD/CAM, additive manufacturing, atrophic jaw, oral rehabilitation, maxillofacial reconstruction, finite element analysis, oncologic reconstruction

## Abstract

**Background/Objectives:** Contemporary patient-specific subperiosteal implants have re-emerged as graftless solutions for oral and maxillofacial rehabilitation, driven by advances in digital planning, CAD/CAM workflows, additive manufacturing, and biomaterial engineering. Their indications have progressively expanded from severely atrophic edentulous jaws to segmental defects, single-tooth replacement, congenital craniofacial anomalies, salvage situations, and oncologic reconstruction. This scoping review aimed to map the current evidence on modern patient-specific subperiosteal implants, focusing on indications, workflow, design principles, materials, outcomes, complications, and maintenance. **Methods:** A scoping review was conducted according to PRISMA-ScR principles to identify clinical studies, case series, case reports, systematic and scoping reviews, technical notes, finite element analyses, in vitro studies, and relevant translational investigations dealing with contemporary custom-made or CAD/CAM subperiosteal implants. The evidence was narratively synthesized according to clinical indication and thematic domains, including full-arch rehabilitation, sectional and single-tooth applications, congenital and post-oncologic defects, rescue indications, biomechanics, material selection, surface response, prosthetic protocols, and complication management. No quantitative meta-analysis was performed because of the scoping design and the substantial heterogeneity of study types, indications, implant systems, outcome definitions, and follow-up durations. **Results:** The final evidence map included 116 records, of which 56 were unique human clinical records with extractable denominators and 60 were biomechanical, in vitro, surface-biology, review, consensus, historical, or conceptual records. Of the 56 unique clinical records, 49 were mapped within the six indication-level clinical sections, while seven were retained as cross-cutting clinical evidence addressing patient-reported outcomes, design-related complications, bone apposition, anchorage strategy, comparative graftless rehabilitation, or reconstructive/prosthetic principles. The six indication-level sections included 52 clinical-record assignments: 15 for full-arch rehabilitation, 13 for segmental or sectional rehabilitation, one for single-tooth rehabilitation, four for congenital or craniofacial indications, 13 for post-oncologic or post-ablative reconstruction, and six for rescue or salvage indications. Because three records addressed more than one indication, these counts represent indication-level assignments rather than mutually exclusive clinical records. Reported survival in most short- to mid-term clinical series was generally high, commonly ranging from 90% to 100%, although lower values of 70–80% were reported in selected longer-term cohorts and survival clearly overestimated clinical success in some studies. Expanding applications include posterior mandibular and maxillary defects, lateral incisor agenesis, cleft-related or syndromic deformities, maxillectomy reconstruction, obturator support, and hybrid rehabilitation with endosseous implants; however, evidence for the indications at the extremes of this spectrum—single-tooth replacement and primary oncologic reconstruction—remains limited to small, largely single-group case series and reports. Soft-tissue events, including dehiscence, mucositis, recession, and framework exposure, were the dominant complications and showed wide variability, with reported recession/exposure rates ranging from approximately 10% in some sectional and full-arch series to as high as 65% in bilateral maxillary cohorts; their clinical significance varied from asymptomatic stable findings to progressive inflammatory complications requiring revision. **Conclusions:** Patient-specific subperiosteal implants represent a promising and increasingly versatile reconstructive option; however, the present findings should be interpreted as evidence mapping rather than as definitive comparative evidence. Their clinical use should remain highly selective, prosthetically driven, and supported by meticulous planning, rigid fixation, soft-tissue management, and structured maintenance. Standardized success criteria, longer follow-up, and comparative prospective studies are required.

## 1. Introduction

Severe alveolar bone atrophy and complex maxillofacial defects remain among the most demanding scenarios in implant-supported oral rehabilitation. In advanced Cawood and Howell class V–VI atrophies, conventional endosseous implant placement may be limited or precluded by insufficient bone volume, unfavorable prosthetic emergence, proximity to anatomical structures, or poor local bone quality. Traditionally, these conditions have been managed through reconstructive procedures such as guided bone regeneration, sinus floor elevation, onlay bone grafting, Le Fort I osteotomy with downgrafting, distraction osteogenesis, or microvascular bone transfer in post-ablative defects. Although these techniques may provide predictable outcomes in selected patients, they are often associated with increased morbidity, prolonged treatment time, higher costs, donor-site complications, and patient reluctance to undergo staged reconstructive pathways [1,2,3].

To reduce surgical invasiveness and treatment duration, several graftless alternatives have been proposed, including short and extra-short implants, tilted implants, pterygoid implants, transnasal implants, and zygomatic implants. These approaches have expanded the therapeutic armamentarium for patients with severe maxillary and mandibular atrophy, but their use is not always feasible. Their indications may be constrained by residual bone anatomy, sinus morphology, zygomatic bone thickness, prosthetic emergence, operator experience, and the risk of procedure-specific complications such as sinusitis, oroantral communication, implant malposition, or difficult prosthetic maintenance [4,5,6,7,8,9]. In addition, these techniques are generally better suited to full-arch rehabilitation and may be unsuitable for localized, segmental, or partially dentate defects requiring closely spaced prosthetic emergences.

Subperiosteal implants were originally introduced in the first half of the twentieth century as an alternative for the rehabilitation of severely resorbed jaws [10]. The first generation of these devices consisted of cast frameworks, commonly made of cobalt-chromium alloys, adapted to the residual alveolar bone after direct bone impression or cast-based fabrication. However, historical subperiosteal implants were burdened by high complication rates, including poor adaptation to the bone, lack of rigid fixation, framework mobility, chronic inflammation, soft tissue dehiscence, epithelial invagination, infection, fistula formation, progressive bone resorption, and eventual implant failure [11,12,13]. With the consolidation of osseointegration-based endosseous implantology, traditional subperiosteal implants were progressively abandoned.

The contemporary reintroduction of subperiosteal implants has been made possible by advances in three-dimensional imaging, virtual surgical planning, computer-aided design and manufacturing, and additive manufacturing. Modern patient-specific subperiosteal implants are no longer passive cast frameworks resting on the alveolar crest, but digitally designed, custom-made, rigidly fixated devices that are planned according to the patient’s anatomy and prosthetic requirements. Cone-beam computed tomography, intraoral scanning, STL/DICOM merging, diagnostic wax-ups, and CAD/CAM workflows allow the fabrication of titanium frameworks that adapt closely to the residual basal bone and are fixed by osteosynthesis screws to stable anatomical buttresses [14,15,16,17,18]. From a biomechanical standpoint, their stability is primarily based on multivectorial rigid fixation and load distribution rather than on classical endosseous osseointegration, making them conceptually closer to patient-specific osteosynthesis devices than to conventional dental implants [19,20,21]. However, contemporary patient-specific subperiosteal implants (PSSI) should not be regarded as a homogeneous implant category. Substantial differences exist among systems in terms of framework geometry, anchorage philosophy, fixation strategy, manufacturing standards, surface characteristics, prosthetic connection, and loading protocol. In particular, the modern PSSI concept introduced a basal bone-anchored, multivectorially fixated framework specifically designed for immediate functional loading in severe maxillary and mandibular atrophy. Recognizing these differences is important, because contemporary devices are not necessarily based on identical biomechanical principles and should not be interpreted as interchangeable solutions.

Initial clinical applications focused mainly on full-arch rehabilitation of severely atrophic edentulous maxillae and mandibles. Several retrospective and prospective reports have described encouraging short- and mid-term survival rates, functional outcomes, and patient satisfaction following rehabilitation with additively manufactured or CAD/CAM-designed subperiosteal implants [17,22,23,24,25,26,27]. Recent systematic reviews have suggested that contemporary digitally planned subperiosteal implants may achieve more favorable survival and complication profiles than historical two-stage devices, although the quality of evidence remains limited by small cohorts, heterogeneous designs, short follow-up, and inconsistent definitions of success and complications [27,28,29,30].

At the same time, the spectrum of indications has rapidly expanded beyond full-arch rehabilitation. PSSI have been proposed for posterior mandibular atrophy, sectional maxillary rehabilitation, isolated molar defects, congenital lateral incisor agenesis, ectodermal dysplasia–cleft syndromes, cleft-related deformities, and hybrid rehabilitations combining subperiosteal and conventional endosseous implants in jaws with heterogeneous residual bone availability [31,32,33,34,35,36,37]. These emerging applications suggest that patient-specific subperiosteal implants should not be regarded solely as a last-resort full-arch solution, but rather as a flexible graftless platform that may be adapted to localized, segmental, congenital, and salvage indications when conventional implants or augmentation procedures are not feasible or have failed.

Another rapidly developing field is post-oncologic and complex maxillofacial reconstruction. PSSI have been used after maxillectomy, in post-ablative midface defects, in patients requiring obturator retention, and in primary or secondary reconstruction combined with soft tissue or free flap procedures [37,38,39,40,41,42,43,44]. In this setting, these devices may serve not only as dental implant supports, but also as patient-specific reconstructive platforms integrating skeletal fixation, prosthetic anchorage, restoration of oral function, and immediate or delayed dental rehabilitation. Innovative anchorage zones, including the pterygoid process, zygomatic arch root, and lateral skull base, have been described for severe midfacial defects where conventional maxillary buttresses are absent or insufficient [43,45].

Despite these promising developments, several unresolved issues remain. The available evidence is heterogeneous; survival is often reported without standardized procedure-specific success criteria, and soft-tissue complications may be interpreted inconsistently across studies. In addition, biomechanical and material-related variables appear to influence stress distribution and complication risk, but much of this evidence remains preclinical or based on finite element models with limited clinical validation [25,46,47,48,49,50,51,52,53,54].

Given this expanding and heterogeneous evidence base, a broader synthesis is needed. Several systematic reviews, scoping reviews, consensus reports, and meta-analyses have appeared between 2024 and 2026 [27,29,30,55,56,57,58,59,60,61,62,63], confirming the renewed interest in contemporary patient-specific subperiosteal implants. However, these publications have generally addressed restricted portions of the field. Some reviews focused primarily on full-arch rehabilitation of severely atrophic edentulous jaws, others on fixed partial restorations or graftless alternatives, while further reports emphasized historical survival trends, consensus-based indications, or biomechanical performance. In addition, recent meta-analytic evidence has highlighted favorable short-term survival but also substantial heterogeneity, overlapping primary studies, limited long-term evidence, and the risk that survival may overestimate true clinical success. Therefore, the knowledge gap is no longer whether modern digitally planned PSSI can be clinically feasible, but how their rapidly expanding indications, heterogeneous outcome definitions, soft-tissue complications, biomechanical principles, surface behavior, and maintenance requirements should be interpreted within a single clinically useful framework.

The present review is intended to address this gap in three specific respects that are not jointly covered by the existing literature: first, by providing a cross-indication map that places full-arch, sectional, single-tooth, congenital, oncologic, and salvage applications within the same comparative framework; second, by explicitly separating survival from success and proposing graded reporting of soft-tissue exposure and procedure-specific success criteria; and third, by integrating biomechanical, material, and surface-biology evidence with prosthetic and maintenance considerations. Accordingly, this scoping review aimed to map the contemporary evidence on CAD/CAM-designed and additively manufactured PSSI, with emphasis on indications, outcomes, complications, design principles, materials, prosthetic protocols, maintenance, and emerging reconstructive applications.

## 2. Materials and Methods

### 2.1. Study Design and Reporting Framework

This scoping review was designed to map the contemporary evidence on CAD/CAM-designed and additively manufactured patient-specific subperiosteal implants used for oral and maxillofacial rehabilitation. The review was conducted and reported in accordance with the Preferred Reporting Items for Systematic Reviews and Meta-Analyses extension for Scoping Reviews (PRISMA-ScR) [64] (Table S1). A written internal protocol was developed before the electronic searches commenced and defined the review question, eligibility criteria, information sources, broad search concepts, study-selection process, data-charting domains, and planned narrative synthesis. However, the protocol was not prospectively registered in a public registry.

A scoping review design was selected because the available literature is highly heterogeneous and includes clinical studies, case series, case reports, technical notes, finite element analyses, in vitro and material studies, systematic and scoping reviews, consensus reports, expert opinions, and historical reports. The purpose was not to answer a narrowly defined comparative effectiveness question or to generate pooled estimates, but to identify, categorize, and critically synthesize the main clinical indications, design principles, outcome patterns, complication profiles, prosthetic strategies, maintenance considerations, and research gaps in this rapidly evolving field.

The review question was structured using a broad Population–Concept–Context framework. The population comprised patients with severe jaw atrophy, localized or segmental edentulism with inadequate bone volume, congenital craniofacial conditions, post-oncologic or acquired maxillofacial defects, or other deformities requiring implant-prosthetic or reconstructive rehabilitation. The concept was the use of contemporary PSSI designed through digital workflows and fabricated using CAD/CAM, selective laser melting, direct metal laser sintering, milling, laser melting, or related additive or subtractive manufacturing techniques. The context included dental, oral, and maxillofacial rehabilitation in the maxilla, mandible, and post-ablative or complex craniofacial defects.

### 2.2. Eligibility Criteria

For evidence mapping, studies were considered eligible when they investigated or provided relevant information on custom-made, patient-specific, CAD/CAM-designed, milled, or additively manufactured subperiosteal implants. Eligible devices included modern rigidly fixated subperiosteal frameworks produced from CT or CBCT data and virtual surgical planning, with or without intraoral scanning, diagnostic wax-up, CAD/CAM design, selective laser melting, direct metal laser sintering, laser melting, milling, or other digitally guided manufacturing processes. Studies evaluating implants used in the maxilla, mandible, or post-ablative maxillofacial defects were eligible when the device was intended to support a fixed prosthesis, removable overdenture, obturator, dental restoration, or implant-borne reconstructive solution.

Eligible evidence included randomized or non-randomized clinical studies, prospective and retrospective cohort studies, case–control studies, case series, technical notes with clinical follow-up, case reports, systematic reviews, scoping reviews, consensus reports, narrative reviews, expert opinions, finite element analyses, in vitro studies, material studies, and preclinical investigations. Case reports and technical notes were retained because of the novelty of the field, but were interpreted separately and used mainly to map indications, workflows, prosthetic applications, complications, and emerging technical principles. Only human clinical studies with extractable denominators were used for survival, success, and complication-rate extraction.

Studies were excluded if they evaluated historical cast subperiosteal implants fabricated through direct bone impression or analog two-stage techniques without contemporary CAD/CAM or additive manufacturing workflows. Studies dealing exclusively with conventional endosseous, zygomatic, pterygoid, transnasal, transosteal, blade implants, or reconstructive plates were excluded unless they included a clearly identifiable subgroup treated with PSSI. Articles were also excluded when the implant type could not be clearly determined, when subperiosteal implant data were inseparable from other implant modalities, or when insufficient information was provided to determine the indication, device characteristics, or outcome of interest. Animal studies were excluded from the clinical synthesis, but were discussed separately when they provided relevant biological or material insights.

No restriction was applied regarding anatomical site, dentition status, prosthetic design, implant material, loading protocol, or follow-up duration. Follow-up duration was extracted and considered when interpreting the maturity of the evidence.

### 2.3. Information Sources and Search Strategy

A comprehensive electronic search was performed in MEDLINE/PubMed, Scopus, Web of Science, Embase, and the Cochrane Library from database inception to 1 May 2026. Additional manual searches were performed by screening the reference lists of all included articles and relevant reviews, consensus reports, technical notes, and systematic reviews. Grey literature and early online publications were considered when they contained sufficient methodological or clinical information.

The search strategy combined controlled vocabulary and free-text terms related to subperiosteal implants, patient-specific implants, CAD/CAM workflows, additive manufacturing, digital dentistry, severe jaw atrophy, maxillary and mandibular reconstruction, and implant-prosthetic rehabilitation. The complete search strategy, including Boolean operators, field tags, truncation, MeSH terms, and connectors, together with database-specific adaptations for PubMed, Scopus, Web of Science, Embase, and the Cochrane Library, is reported in Supplementary Table S2.

The Cochrane Library search retrieved zero records before deduplication; this was considered consistent with the limited trial-based and review-level evidence available for contemporary patient-specific subperiosteal implants.

No date restriction was applied during the initial search. During study selection, articles were categorized as historical or contemporary according to implant concept and manufacturing workflow.

### 2.4. Study Selection and Management of Overlapping Evidence

All records retrieved from electronic searches were imported into reference management software, and duplicates were removed before screening. Study selection was performed independently by two reviewers. Titles and abstracts were first screened to exclude clearly irrelevant records, and the full texts of potentially eligible articles were then assessed against the predefined inclusion and exclusion criteria. Inter-reviewer agreement for full-text eligibility assessment was excellent, with a Cohen’s kappa coefficient of 0.82. Disagreements were resolved by discussion or, when required, by consultation with a third reviewer. Reasons for exclusion at the full-text stage were recorded and summarized in the PRISMA-ScR flow diagram [64].

When multiple publications appeared to describe overlapping patient populations or the same clinical cohort, quantitative data were extracted only once, prioritizing the publication with the largest sample size, longest follow-up, and most complete outcome reporting. Companion publications were retained for qualitative discussion only when they provided non-duplicative information on surgical technique, prosthetic protocol, implant design, radiological assessment, soft tissue response, or complication management. This approach was adopted to reduce the risk of double counting while preserving clinically relevant technical information. To further clarify the distinction between unique clinical records and indication-level assignments, each human clinical record with extractable denominators was assigned to one primary category and, when appropriate, to one or more secondary indication categories. Therefore, the numbers reported in the indication-level sections represent clinical-record assignments, not mutually exclusive unique records. Records addressing more than one indication were counted once in the total number of clinical records and once per relevant indication in the cross-indication map. Clinical records whose main contribution concerned patient-reported outcomes, design-related complications, bone apposition, anchorage strategy, comparative graftless rehabilitation, or reconstructive/prosthetic principles were retained as cross-cutting clinical evidence.

### 2.5. Data Charting and Outcomes

Data were charted independently by two reviewers using a predefined data-charting form. For clinical studies, extracted variables included bibliographic details, country, study design, number of patients and implants, demographics, anatomical site, dentition status, treatment indication, atrophy or defect classification when available, previous failed treatments, oncologic or congenital background, radiotherapy history, smoking status, systemic risk factors, implant material, manufacturing method, software workflow, implant design, number and position of abutments, prosthetic connection, fixation screw configuration, anatomical anchorage zones, framework thickness, surface characteristics, adjunctive soft-tissue procedures, loading protocol, provisional and definitive prosthetic design, follow-up duration, survival, success, complications, reinterventions, implant removal, radiological findings, and patient-reported outcomes.

For finite element, in vitro, and material studies, extracted variables included model type, anatomical region, simulated implant design, material properties, loading conditions, boundary conditions, validation strategy, stress distribution, displacement, failure thresholds, biological endpoints, and the authors’ main biomechanical or biological conclusions. These studies were not pooled with clinical studies, but were used to support interpretation of design, anchorage, material selection, surface biology, and possible mechanisms of biological or mechanical complications.

The main clinical outcome was implant survival, defined as the presence of the subperiosteal implant in situ and in function at the last reported follow-up. Prosthetic or rehabilitation survival was recorded when available. Because universally accepted success criteria for contemporary custom-made subperiosteal implants have not yet been established, success was extracted only when explicitly defined or clearly reported by the original authors. When success was not defined, we did not reclassify survival as success. Instead, such cases were described as functional survival without major failure when the implant remained in situ, supported the intended rehabilitation, showed no clinical mobility, and did not require removal or major surgical revision.

For interpretive purposes, true clinical success was considered broader than survival and was understood to require, when reported, stable prosthetic function, absence of implant mobility or major revision, absence of persistent infection or suppuration, absence of progressive or symptomatic framework exposure, acceptable soft-tissue health, manageable maintenance burden, and patient-reported functional benefit. Because these domains were inconsistently reported across studies, they were not used to generate a uniform post hoc success rate, but were charted narratively whenever available.

Secondary outcomes included biological, mechanical, and prosthetic complications; soft tissue response; radiological stability; loading protocol; patient-reported outcomes; and maintenance-related findings. Biological complications included wound dehiscence, mucosal recession, framework exposure, mucositis, infection, suppuration, sinus complications, persistent pain, or delayed non-suppurative inflammatory episodes. Mechanical complications included fixation screw loosening or fracture, framework fracture, prosthetic screw loosening, prosthetic fracture, crown decementation, loss of stability, or implant mobility not primarily attributable to infection. Framework exposure was interpreted according to severity, symptoms, progression, and need for treatment whenever these details were available.

### 2.6. Evidence Appraisal and Synthesis

Given the scoping nature of this review and the marked heterogeneity of the included literature, a formal risk-of-bias assessment was not performed. Instead, each record was interpreted narratively according to study design, sample size, follow-up duration, clarity of the clinical indication, completeness of outcome reporting, definition of survival and success, complication reporting, and presence of extractable denominators. Case reports and technical notes were considered useful for mapping emerging indications, workflows, reconstructive strategies, and technical refinements, but were not interpreted as evidence of clinical effectiveness. Similarly, finite element analyses, in vitro studies, and material studies were used as mechanistic evidence to support discussion of design principles, surface biology, and material selection, but were not combined with clinical outcome evidence. We further assessed author-group non-independence at the clinical-record level. For this purpose, a record was classified as author-group evidence when at least one co-author of the present review appeared among the authors of the original clinical publication; all other records were classified as independent. Overall, 19 of the 56 unique human clinical records with extractable denominators (33.9%) included one or more co-authors of the present review. However, non-independence was not evenly distributed across indications. Author-group records accounted for 5 of 15 full-arch assignments (33.3%), 4 of 13 segmental/sectional assignments (30.8%), 1 of 1 single-tooth assignment (100%), 1 of 4 congenital/craniofacial assignments (25.0%), 5 of 13 post-oncologic/post-ablative assignments (38.5%), and 1 of 6 rescue/salvage assignments (16.7%). In addition, 2 of 7 cross-cutting clinical records (28.6%) were classified as author-group evidence. Importantly, within the post-oncologic/post-ablative category, the primary reconstruction signal was less independent than the broader category-level figure suggests, because the principal primary oncologic or midface reconstruction reports were all produced by the author group. Thus, the indications supported by the thinnest evidence, particularly single-tooth replacement and primary oncologic reconstruction, also showed the greatest author-group non-independence. To mitigate interpretive bias, conclusions resting predominantly on the authors’ own work are explicitly identified as preliminary, non-independent, or hypothesis-generating; preliminary or non-primary publications by the author group are not treated as load-bearing evidence; and reviews co-authored by members of this author list are cited as concordant opinion rather than as independent confirmation.

No formal downgrading of individual records was performed, because this scoping review did not include a formal risk-of-bias assessment or certainty-of-evidence grading. However, author-group non-independence was incorporated into the narrative synthesis as an informal sensitivity consideration: conclusions supported predominantly or exclusively by author-group records were checked against the presence or absence of independent clinical evidence and, when independent replication was lacking, were framed as preliminary, non-independent, and hypothesis-generating rather than as established evidence. No record was excluded solely because of author overlap, but author-group records were not used as the sole basis for strong or practice-defining conclusions.

A qualitative synthesis was organized according to clinical indication, anatomical site, prosthetic application, and evidence type. Clinical studies were grouped into full-arch rehabilitation of severely atrophic jaws, sectional or partial edentulism, single-tooth applications, congenital and craniofacial indications, hybrid rehabilitations combining subperiosteal and endosseous implants, post-oncologic and maxillofacial reconstructive applications, and salvage or rescue indications. Biomechanical, in vitro, material, and surface-related studies were synthesized separately to avoid mixing clinical outcome evidence with mechanistic or preclinical evidence.

No quantitative meta-analysis was performed because the objective of this scoping review was to map the breadth, nature, and distribution of the available evidence rather than to estimate a pooled treatment effect. Quantitative pooling was not appropriate because of substantial heterogeneity in study design, anatomical indication, implant system, prosthetic protocol, loading strategy, reporting level, outcome definition, and follow-up duration. Survival, success, and complication data were therefore summarized descriptively when extractable denominators were available.

When possible, outcomes were summarized separately at the patient, implant, abutment, screw, prosthesis, and rehabilitation levels. This distinction was considered essential because several studies reported bilateral implants, multiple abutments, coupling elements, fixation screws, or prosthetic units within the same patient. The overall maturity of the evidence base was discussed narratively rather than graded formally, with particular caution applied to high survival rates from small cohorts with short follow-up and to indirect comparisons with historical devices, conventional endosseous implants, zygomatic implants, or bone augmentation techniques.

## 3. Results

### 3.1. Study Selection

**Figure 1:**
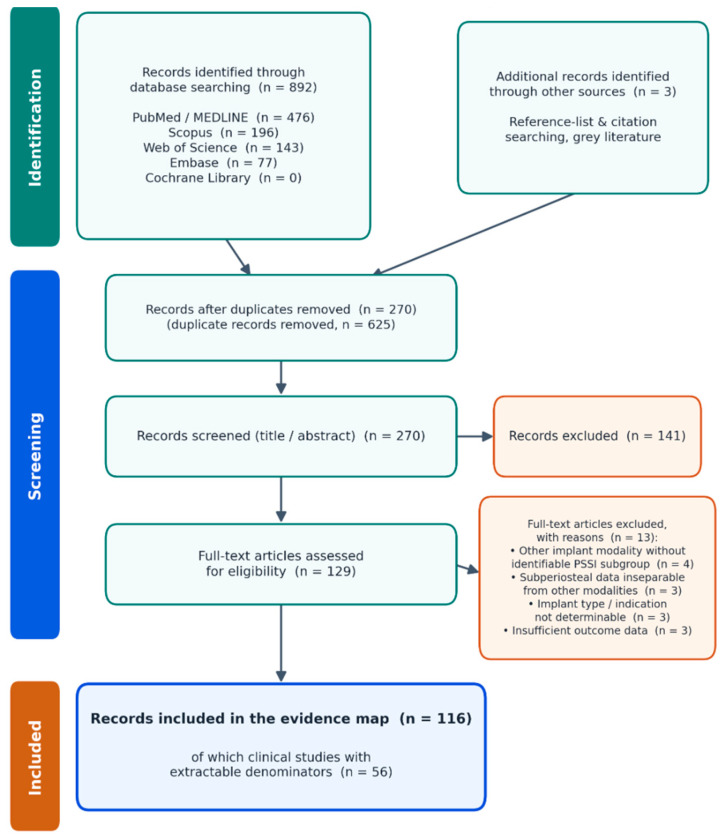
PRISMA-ScR flow diagram of study selection.

The study selection process is reported in the PRISMA-ScR flow diagram [Figure 1]. Overall, the final evidence base comprised 116 unique records eligible for evidence mapping. Of these, 56 were unique human clinical records with extractable denominators, including case reports and technical notes with clinical follow-up, from which survival, success, and complication data were charted; the remaining 60 records consisted of biomechanical finite element analyses, in vitro and surface-biology studies, systematic, scoping and narrative reviews, consensus reports, expert commentaries, and historical or conceptual records. Of the 56 unique clinical records, 49 were mapped within the six indication-level clinical sections, while seven were retained as cross-cutting clinical evidence because their main contribution concerned patient-reported outcomes, design-related complications, bone apposition, anchorage strategy, comparative graftless rehabilitation, or reconstructive/prosthetic principles. The six indication-level sections contained 52 clinical-record assignments because three records were assigned to more than one indication category. The complete record-to-category mapping is reported in Supplementary Table S3.

The included literature was highly heterogeneous in terms of study design, anatomical indication, implant concept, prosthetic protocol, and outcome reporting. The evidence ranged from historical reports and complications of first-generation subperiosteal implants to modern digitally planned, CAD/CAM or additively manufactured patient-specific devices. Historical and legacy implant evidence was represented by reports describing long-term maintenance or complications of older subperiosteal frameworks [65,66,67,68], whereas contemporary clinical evidence focused on custom-made titanium, PEEK, or hybrid patient-specific frameworks manufactured through digital workflows [17,18,22,23,27,28,30,31,36,55,56,69].

The included records were not limited to full-arch rehabilitation of severely atrophic edentulous jaws. A substantial proportion addressed segmental, sectional, congenital, post-oncologic, post-infective, reconstructive, and rescue indications. These included posterior mandibular and posterior maxillary segmental rehabilitation [25,31,33,70], fixed partial restorations [55], single-tooth or anterior esthetic rehabilitation in lateral incisor agenesis [35], congenital craniofacial anomalies such as EEC syndrome or cleft-related deformities [71,72], oncologic and post-maxillectomy reconstruction [15,37,38,39,40,57,73], post-infective defects including post-COVID mucormycosis [74,75,76], and salvage situations after failure of conventional, zygomatic, or pterygoid implants [32,77,78,79].

In parallel, a large body of mechanistic and indirect evidence was identified. Biomechanical studies used finite element analysis or experimental mechanical validation to evaluate framework geometry, anchorage distribution, material properties, screw configuration, fatigue behaviour, and prosthetic load transfer [19,20,26,49,50,51,53,54,58,80,81,82,83,84,85,86]. Material and surface studies investigated titanium, Ti6Al4V, PEEK, PEKK, CFR-PEEK, NiTi, anodized or treated titanium surfaces, osteoblast-like cell response, fibroblast behaviour, and bacterial adhesion [87,88,89,90,91,92,93,94]. These studies were considered supportive mechanistic evidence and were not pooled with clinical outcomes.

### 3.2. General Characteristics of Included Studies

The included records were organized into a general evidence map according to year, country, study type, population or indication, anatomical site, number of patients, number of implants, follow-up, and main outcome. The evidence map showed a clear chronological shift from historical cast subperiosteal implants to digitally planned patient-specific frameworks. Older-generation devices were generally described in relation to long-term complications, maintenance of legacy implants, or late removal, whereas modern studies emphasized CBCT-based planning, prosthetically driven reverse engineering, CAD/CAM design, additive manufacturing, rigid screw fixation, and the use of stable anatomical buttresses.

Clinical evidence included fully edentulous, partially edentulous, congenital, reconstructive, and salvage populations. Full-arch rehabilitation was mainly reported in severely atrophic maxillae and mandibles [17,18,22,23,24,30,69,95,96,97,98], whereas more recent studies increasingly addressed sectional defects [31,33,36,55,70], single-tooth indications [35], congenital craniofacial deformities [71,72,77,99], hybrid rehabilitations [32], post-oncologic reconstruction [15,37,38,40,41,42,43,57,73,100,101], and rescue scenarios after failure of conventional or zygomatic implants [32,72,76,77,78,79,102,103]. Because these indications differ substantially in anatomy, prosthetic goals, risk profile, and expected outcomes, they were synthesized separately in the following sections rather than aggregated into a single clinical outcome category. These sections should therefore be interpreted as an indication map rather than as mutually exclusive groups of studies. When a publication addressed more than one clinical context, it was assigned to all relevant indication categories but counted only once in the total number of unique clinical records.

The evidence map also included systematic reviews, scoping reviews, and consensus reports. These studies summarized the available clinical outcomes, reported high short-term survival of modern digital subperiosteal implants, and emphasized soft-tissue complications, exposure, mucositis, infection, and the lack of standardized success criteria as major limitations of the field [27,28,29,30,55,56,57,59,60]. Two recent meta-analyses are particularly germane to the limited long-term evidence. Gellrich et al. pooled survival across studies and confirmed favourable short-term but uncertain long-term outcomes [62], while Cosola et al., searching up to November 2025, analysed eleven studies (268 patients, 369 implants) and reported a pooled short-term survival of 97.8% for follow-up ≤3 years that declined to 54.1% in the longest (6-year) cohort, with soft-tissue exposure and dehiscence as the dominant complications and the main cause of late failure; the authors concluded that survival alone overestimates clinical success [63]. However, these reviews also showed substantial overlap in included primary studies, heterogeneous inclusion criteria, and limited feasibility for meta-analysis because of variable definitions of implant survival, success, complications, and follow-up duration.

### 3.3. Methodological Characteristics and Maturity of the Evidence

The overall maturity of the evidence was limited by the predominance of low-level designs. Of the 116 records, 56 were unique human clinical records with extractable denominators and 60 were non-clinical, review, biomechanical, in vitro, historical, or conceptual records. Among the 56 clinical studies, 26 (46%) were case reports or technical notes, 28 (50%) were retrospective or prospective cohorts and case series, and only 2 (4%) were comparative studies, of which one was a randomized controlled trial [104]. Only about 10 clinical studies (18%) reported follow-up beyond three years and only 5 (9%) beyond five years, and prospective or multicentre designs remained uncommon. In addition, approximately 35% of the clinical studies included one or more co-authors of the present manuscript; excluding these non-independent studies would not change the overall conclusion that full-arch rehabilitation is the most mature indication, but it would further reduce the evidence depth for several emerging applications, particularly sectional/hybrid, single-tooth, congenital/craniofacial, and primary oncologic indications.

## 4. Evidence by Clinical Indication and Evidence Depth

### 4.1. Established Indication: Full-Arch Rehabilitation of Severely Atrophic Edentulous Jaws

This section included 15 indication-level clinical-record assignments addressing full-arch rehabilitation of severely atrophic edentulous jaws [Table 1]. This was the most frequently reported and most mature contemporary indication for PSSI, particularly in patients with Cawood and Howell class V–VI resorption who are unwilling or unsuitable to undergo extensive bone augmentation. In this setting, modern subperiosteal implants have been proposed as graftless alternatives to sinus floor elevation, onlay grafting, guided bone regeneration, Le Fort I downgrafting, zygomatic implants, and other complex reconstructive protocols. The rationale is not simply to replace endosseous implants, but to provide a patient-specific framework capable of obtaining immediate mechanical stability through rigid screw fixation to stable basal bone and anatomical buttresses.

The available clinical literature suggests encouraging short- and mid-term outcomes, although the evidence remains heterogeneous.

Across full-arch studies, short- and mid-term survival was generally favorable, particularly in modern CAD/CAM-designed or additively manufactured devices. Early and contemporary series reported high functional survival in severely atrophic jaws, while prospective and multicenter PSSI studies showed improvements in patient-reported outcomes and radiographic stability in the short term [17,18,22,23,24,25,69,95,105]. However, results were not uniformly positive. Several studies highlighted the importance of passive fit, soft-tissue management, and infection control, with failures or reduced success mainly related to insufficient adaptation, recurrent infection, framework exposure, mucosal recession, or progressive inflammatory complications [18,23,46,97,106,107]. Systematic reviews were consistent with this interpretation, reporting high short-term survival but substantial heterogeneity, limited follow-up, and non-negligible rates of exposure or soft-tissue complications [27,28,29,56].

Taken together, the evidence on full-arch rehabilitation suggests that modern CAD-CAM and additively manufactured PSSI can achieve high short- and mid-term survival in severely atrophic edentulous jaws. However, their complication profile differs from that of conventional endosseous implants. Their success depends less on classic fixture osseointegration and more on passive fit, rigid multivectorial fixation, prosthetically driven abutment positioning, soft-tissue thickness, biofilm control, and long-term maintenance. Long-term studies remain limited, and caution is required when extrapolating short-term survival data to durable biological success.

### 4.2. Emerging Indication with Moderate Evidence Depth: Segmental and Sectional Rehabilitation

This section included 13 indication-level clinical-record assignments addressing segmental, sectional, hybrid, or partially edentulous rehabilitation [Table 2]. A major evolution in the field is the progressive shift from full-arch salvage rehabilitation toward segmental and sectional indications. This is clinically important because many patients with severe jaw atrophy are not completely edentulous and do not require full-arch reconstruction. Instead, they may present localized posterior maxillary or mandibular bone deficiency, partially dentate arches, or isolated edentulous sectors where conventional implant placement would require grafting, sinus augmentation, or nerve-related procedures. In these cases, modern subperiosteal implants may function as site-specific graftless devices rather than full-arch substitutes.

The posterior mandible is one of the most relevant sectional indications. Available reports suggest that PSSI may be useful when residual bone height above the inferior alveolar nerve is insufficient for conventional implants and when vertical augmentation, short implants, or nerve lateralization are considered risky or unpredictable [31,34,70].

The posterior maxilla is another key indication, particularly when sinus lift is refused or contraindicated and zygomatic or pterygoid implants are not prosthetically appropriate for a limited posterior defect. The available technical and clinical evidence suggests that careful palatal incision design, crestal abutment housing, basal buttress fixation, and selective soft-tissue thickening may be important factors in reducing exposure risk in sectional maxillary rehabilitation [33,36].

The scoping review by Ruiz-Rincón et al. specifically evaluated PSSI supporting fixed partial restorations. Across seven studies, 96 patients and 121 implants were included, with a weighted mean survival rate of 99.17%, biological complications in 4.13%, and mechanical complications in 7.44% over follow-up periods ranging from one to four years [55]. Although these results are encouraging, the authors appropriately emphasized the small sample size, heterogeneity of the included studies, absence of comparative trials, and lack of information on important variables such as opposing dentition and gingival biotype. Their review supports the concept that fixed partial rehabilitation is an emerging indication, but also confirms that robust indication-specific evidence is still lacking.

Finally, the hybrid rehabilitation concept further expands the segmental indication. Vaira et al. reported 14 patients treated with 20 custom-made subperiosteal implants and 48 conventional endosseous implants within the same jaw, achieving 100% survival for both implant systems and 100% rehabilitation survival at a mean follow-up of 22.1 months [32]. This approach is clinically rational in patients with heterogeneous residual bone availability: endosseous implants are used where sufficient native bone remains, while subperiosteal implants are reserved for critically deficient sectors, because conventional implants are supported by stronger long-term evidence, more standardized protocols, and more widely validated outcome expectations.

Overall, the evidence on segmental and sectional rehabilitation indicates that PSSI are increasingly being used not only as salvage full-arch devices, but also as site-specific graftless solutions for localized severe atrophy, congenital agenesis, and complex partially dentate patients. This represents one of the most important conceptual changes in the modern subperiosteal implant literature.

### 4.3. Exploratory Indication with Low Evidence Depth: Single-Tooth Rehabilitation

This section included one indication-level clinical-record assignment addressing single-tooth rehabilitation with PSSI. Single-tooth rehabilitation therefore represents one of the most recent and most exploratory extensions of PSSI. Traditionally, subperiosteal implants were conceived for severely atrophic edentulous jaws, mostly in elderly patients or in complex full-arch rehabilitations.

The application of PSSI to single-tooth defects therefore marks a substantial shift in indication, moving from broad salvage frameworks to highly localized, prosthetically driven devices designed to solve focal bone deficiency in esthetically demanding areas.

The most directly relevant clinical evidence in this field is the case series by Roy et al., who reported the use of PSSI for maxillary lateral incisor agenesis in young patients with severe localized alveolar deficiency and previous failure of conventional endosseous implant treatment [35]. This indication is particularly relevant because lateral incisor agenesis is frequently associated with a narrow or deficient alveolar crest, limited buccopalatal bone volume, and high esthetic expectations. In such patients, conventional management may include orthodontic space closure, resin-bonded bridges, fixed prosthodontics, or guided bone regeneration followed by endosseous implant placement. However, when previous regenerative or endosseous implant strategies fail, or when the residual ridge anatomy does not allow predictable implant placement without extensive morbidity, a customized subperiosteal implant may provide a site-specific alternative.

In the series by Roy et al., 12 patients received 16 PSSI for lateral incisor agenesis, with a mean follow-up of 48 months and a reported implant survival of 100% [35]. The authors also reported favorable peri-implant soft tissue conditions, absence of implant exposure or gingival recession, a high mean pink esthetic score, and only minor prosthetic complications consisting of two crown recementations after three years. These outcomes suggest that, in highly selected cases, PSSI may be adapted to single-tooth anterior rehabilitation, provided that implant design, abutment emergence, soft-tissue contour, and esthetic integration are carefully planned.

Nevertheless, this indication requires particular caution. Unlike elderly edentulous patients, young patients with single-tooth defects have long life expectancy, high esthetic expectations, and a greater cumulative risk of future revision. The biological and mechanical behavior of very small subperiosteal frameworks over decades remains unknown. Moreover, the threshold for using a relatively novel implant concept should remain high when less invasive and more established options, such as resin-bonded prostheses or orthodontic space closure, are feasible. Therefore, single-tooth PSSI should currently be interpreted as a selected rescue or alternative strategy for localized severe bone deficiency, rather than as a routine replacement for conventional anterior implant or prosthetic protocols.

### 4.4. Exploratory Indication with Low Evidence Depth: Congenital and Craniofacial Rehabilitation

This section included four indication-level clinical-record assignments addressing congenital, syndromic, or craniofacial indications [Table 3]. These indications represent a distinct category from single-tooth rehabilitation. In these patients, the clinical problem is not simply the absence of one tooth or a localized alveolar defect, but the coexistence of altered skeletal anatomy, previous reconstructive procedures, cleft-related deformity, syndromic craniofacial growth disturbance, hypodontia, scarred tissues, and limited residual bone volume. These conditions often make conventional endosseous implant placement difficult or unpredictable, while repeated grafting may be associated with substantial morbidity, uncertain resorption, or poor patient acceptance.

The case report by De Riu et al. on ectrodactyly–ectodermal dysplasia–cleft syndrome is the clearest example of this indication [71]. The patient had a history of cleft repair, surgically assisted palatal expansion, iliac crest grafting, maxillary advancement with onlay grafting, rhinoplasty, and persistent severe maxillary hypoplasia and alveolar atrophy. Despite multiple previous reconstructive procedures, the residual bone volume remained insufficient for conventional endosseous implants. Two PSSI were planned using CBCT, digital impressions, and prosthetic wax-up, with fixation to the nasomaxillary and maxillomalar pillars. Immediate loading was performed, and the definitive prosthesis was delivered after soft-tissue conditioning. At 18 months, the implants were stable, with no exposure, bleeding on probing, soft-tissue complication, or prosthetic problem. This report is important because it shows that PSSI may offer a tailored rehabilitation option in syndromic patients with complex craniofacial anatomy and limited conventional alternatives.

Other reports support the broader concept that PSSI may be useful in congenital or craniofacial deformities. Ângelo and Ferreira described a patient with dental agenesis and previous failed mandibular all-on-6 rehabilitation due to peri-implantitis, treated with bimaxillary custom subperiosteal implants incorporating both subperiosteal and endosseous support concepts [77]. Although this report is not limited to a single congenital syndrome, it is relevant because it demonstrates the use of customized frameworks in a patient with agenesis, complex rehabilitation needs, and previous implant failure. Similarly, Wirth et al. reported a patient with unrepaired cleft palate, multiple failures of conventional and zygomatic implants, chronic sinusitis, and severe maxillary deformity, rehabilitated with PSSI combined with residual tuberosity implants to support an implant-retained obturator [72]. This case reinforces the potential role of subperiosteal implants as salvage devices in congenital craniofacial anatomy, particularly when standard endosseous and zygomatic solutions have failed or are anatomically unfavorable.

Debortoli et al. described a young patient with severe skeletal class III deformity, maxillary transverse deficiency, and compromised dentition treated with Le Fort I osteotomy and PSSI in the same reconstructive-prosthetic pathway [99]. Although this is better classified as an orthognathic-assisted rehabilitation rather than a strictly congenital syndrome, it is relevant to the craniofacial indication because it shows how subperiosteal implants may be integrated into skeletal repositioning procedures to reduce prosthetic compensation and optimize facial and occlusal outcomes. This type of indication remains anecdotal, but it broadens the conceptual role of subperiosteal implants beyond static rehabilitation of atrophic bone.

Compared with full-arch atrophy, congenital and craniofacial indications raise additional concerns. Patients may be young, have long-term maintenance needs, and present altered soft-tissue quality due to scars, cleft surgery, previous grafts, or syndromic ectodermal abnormalities. In addition, prosthetic planning is often more complex because esthetics, speech, lip support, occlusal plane, facial projection, and hygiene accessibility must be considered simultaneously. However, the available evidence consists only of four case reports, with limited follow-up and no comparative data. Therefore, PSSI in congenital and craniofacial patients should currently be interpreted as hypothesis-generating and highly selected individualized reconstructive-prosthetic attempts, rather than as an established indication. Their use may be considered only when conventional implantology, bone grafting, or zygomatic/pterygoid approaches are not feasible, have failed, or would not provide a prosthetically acceptable outcome, and their long-term predictability requires independent validation.

### 4.5. Exploratory Indication with Low Evidence Depth: Post-Oncologic and Maxillectomy Reconstruction

This section included 13 indication-level clinical-record assignments addressing post-oncologic, post-ablative, post-infective, maxillectomy, MRONJ-related, benign tumor-related, or complex reconstructive applications [Table 4]. Post-oncologic and maxillectomy reconstruction represents one of the most distinctive emerging indications for PSSI. In this setting, the device may function as a reconstructive platform rather than as a purely dental implant, integrating skeletal fixation, prosthetic anchorage, obturator retention, soft-tissue support, and immediate or delayed oral rehabilitation.

The conceptual foundation was established by Gellrich et al., who described customized digitally engineered solutions for fixed dental rehabilitation in severe bone deficiency and resected jaws [15,113]. These patient-specific devices were designed from three-dimensional imaging and prosthetic wax-up, with skeletal anchorage and prosthetic connections integrated into a single digital plan. Vosselman et al. subsequently reported a patient-specific subperiosteal zygoma implant for a large maxillary defect after oncologic treatment, using the residual zygomatic structures to support an obturator and rapidly improve speech and swallowing [73]. Cebrián-Carretero et al. reported customized subperiosteal titanium maxillary implants in four patients with maxillary defects after oncologic resection, using virtual surgical planning, STL models, CAD/CAM titanium mesh, and fixed prosthetic rehabilitation [38]. These studies showed that patient-specific subperiosteal frameworks could be used not only in atrophic jaws, but also in surgically altered maxillofacial anatomy.

A major development is the use of subperiosteal implants in primary reconstruction. De Riu et al. reported a case of total maxillectomy reconstructed with a PSSI and temporal muscle flap in an elderly patient who was not a candidate for free flap reconstruction [41]. The implant was designed and manufactured in a short time, fixed to residual maxillary and zygomatic structures, and used to support both midfacial soft tissues and a prosthetic obturator. Although a fistula occurred due to temporal fascia necrosis, no implant-related complication was reported at six months. More recently, De Riu et al. described primary reconstruction of a total maxillectomy defect using a fibula free flap associated with a PSSI [39]. In that case, the implant was integrated into a digital plan including craniofacial CT, dental impressions, wax-up, lower-limb angio-CT, fibula cutting guides, and Ti6Al4V DMLS manufacturing. The abutments were submerged during adjuvant radiotherapy and uncovered later, with definitive prosthetic rehabilitation at six months and no complications at two years.

The strongest clinical signal in this area comes from the nine-case series by De Riu et al., in which three-dimensionally printed PSSI were used for primary maxillary reconstruction during oncologic surgery [37]. The implants acted simultaneously as osteosynthesis plates and prosthetic abutment platforms. Despite the complexity of the cases, including malignant tumors, free flaps, local flaps, and adjuvant radiotherapy or chemoradiotherapy in several patients, all implants were positioned and loaded, with no infection, mobility, or prosthetic complications during a mean follow-up of 13.7 months. One flap necrosis was not directly related to the implant, one limited exposure was reported, and one patient developed severe mucositis around transmucosal abutments during radiotherapy. This series is highly relevant because it demonstrates that patient-specific subperiosteal implants may be incorporated into oncologic resection and reconstruction planning from the beginning, rather than being used only as secondary rehabilitation after defect stabilization.

Other reports support similar concepts. Segna et al. described one-step primary complete rehabilitation after maxillectomy for a benign tumor in a young patient, combining free fibula flap, PSSI, orbital mesh, and immediate prosthetic rehabilitation [40]. Frias et al. used a custom maxillary subperiosteal implant to retain an immediate surgical obturator in an elderly patient with an oro-antral fistula and compromised residual dentition, later converting the rehabilitation to an implant-retained removable obturator [42]. Machine and Nadjmi described maxillary reconstruction after hemimaxillectomy using a PSSI and overdenture, selecting a removable prosthetic solution to improve hygiene under a bulky flap [101]. These studies illustrate that the prosthetic endpoint may vary according to defect anatomy, soft-tissue volume, hygiene accessibility, and patient frailty: fixed prostheses are not always the optimal solution.

The most complex oncologic and midfacial defects may require unconventional anchorage zones. Gellrich et al. reported innovative landing zones for one-piece, rigidly fixated PSSI, extending anchorage to the lateral skull base, zygomatic arch root, and pterygoid process when traditional buttresses were insufficient [43]. These designs were applied in post-ablative, traumatic, cystic, and severely atrophic defects, with generally favorable outcomes but also at least one case requiring removal for chronic pain and infection. This evidence supports the feasibility of extended anchorage, but also underscores the need for caution because these cases are highly individualized and depend heavily on surgical expertise, tissue quality, and preoperative biomechanical planning.

Systematic evidence remains limited. Oliveira et al. identified only six studies and 31 patients treated with PSSI after maxillofacial oncologic resection, with no implant failures reported over follow-up periods ranging from six to 26 months [57]. The results were clinically encouraging, but the evidence consisted only of case reports and case series. Therefore, while patient-specific subperiosteal implants appear promising in post-oncologic reconstruction, they cannot yet be considered supported by high-level evidence.

Overall, in oncologic reconstruction, PSSI are evolving from dental rehabilitation devices into multifunctional reconstructive platforms. They may allow simultaneous or staged integration of skeletal reconstruction, prosthetic planning, obturator retention, soft-tissue support, and oral rehabilitation. Their use is particularly attractive in maxillectomy defects, irradiated or scarred tissues, poor candidates for extensive secondary surgery, and cases in which immediate restoration of speech, swallowing, and facial projection is clinically important. However, radiotherapy, mucositis, tissue fragility, flap bulk, infection risk, and hygiene access remain critical determinants of success.

### 4.6. Exploratory Indication with Low Evidence Depth: Rescue and Salvage Indications

This section included six indication-level clinical-record assignments addressing rescue or salvage use of PSSI after failure or impracticability of conventional, regenerative, zygomatic, pterygoid, reconstructive, or prosthetic approaches [Table 5]. Because this evidence is entirely case-report based and generally short term, rescue or salvage use should be regarded as a descriptive and exploratory application rather than as a validated indication.

This indication is clinically important because many candidates for subperiosteal implants present after multiple failed treatments, with reduced residual bone, scarred soft tissues, sinus complications, or reluctance to undergo further grafting procedures.

Several studies describe subperiosteal implants after failure of conventional endosseous implants or bone regeneration. Revuelta-Cortés et al. reported the use of a CAD/CAM PSSI in a severely atrophic maxilla after implant failure and peri-implantitis, emphasizing the role of the digital workflow as a rescue strategy when residual bone is compromised [79]. Ângelo and Ferreira reported bimaxillary custom subperiosteal implants in a patient with agenesis and previous failed mandibular all-on-6 rehabilitation due to peri-implantitis [77].

Subperiosteal implants have also been proposed after failure or impracticability of other graftless approaches. Vaira et al. described a hybrid rehabilitation case in which a planned pterygoid implant could not be placed because of insufficient primary stability; a PSSI was subsequently planned and used as a secondary graftless solution [32]. Cardoso and Grillo reported a patient with complications and sequelae after bilateral zygomatic implants, managed by removal of the zygomatic implants and placement of a PSSI, with improvement in quality of life after more than one year of follow-up [78]. Wirth et al. described a patient with unrepaired cleft palate, multiple failed endosseous and zygomatic implants, chronic sinusitis, and severe maxillary bone loss, rehabilitated with PSSI and residual tuberosity implants to support an obturator [72]. These cases support the concept that subperiosteal implants may be useful when other graftless solutions fail or are anatomically unsuitable.

Post-infective and acquired defects represent another salvage setting. Parras-Hernández et al. [114] described a personalized subperiosteal implant-supported obturator for rehabilitation of severe maxillary sequelae after rhino-orbit-cerebral mucormycosis, in a patient in whom fibula free flap reconstruction had failed. This report further supports the role of patient-specific subperiosteal implants as salvage platforms in highly complex post-infective defects when conventional reconstructive approaches are no longer feasible or have already failed.

Failure and removal considerations are also relevant. Manor et al. analyzed the removal of unconventional implants over an 11-year period and highlighted the complexity of managing failed nonconventional devices [103]. This reinforces the need to consider explantability, access to fixation screws, modularity, and future salvage options during the initial design phase of PSSI. In complex patients, the question should not only be whether the implant can be placed and loaded, but also how it can be monitored, revised, partially modified, or removed if biological or mechanical complications occur.

Taken together, rescue and salvage cases show that PSSI may offer a valuable option after failure of conventional, regenerative, or graftless treatments. However, these cases are also among the most biologically and surgically demanding. Scar tissue, previous infection, sinus disease, altered anatomy, reduced vascularity, smoking, poor hygiene, radiotherapy, or previous peri-implantitis may increase the risk of complications. Therefore, rescue use should be based on careful patient selection, realistic counseling, soft-tissue optimization, prosthetically driven planning, and a design strategy that anticipates future maintenance or revision.

## 5. Biomechanical and Design Principles

### 5.1. Fixation-Based Biomechanics Rather than Osseointegration-Based Stability

A central conceptual difference between contemporary PSSI and conventional endosseous implants is that primary stability is mainly fixation-based rather than fixture-based [Table 6]. Modern PSSI behave more like patient-specific osteosynthesis devices: stability depends on passive adaptation to the residual bone surface, rigid screw fixation, and load distribution across stable buttresses rather than on immediate endosseous anchorage along a threaded fixture [14,15,113,115].

In atrophic maxilla models, stresses are mainly concentrated around the arms, fixation screws, abutment–framework junctions, and areas subjected to bending moments, rather than being uniformly distributed along an intraosseous fixture surface [19,81,82]. Therefore, the clinical reliability of these devices depends on passive fit, screw number and distribution, screw diameter and length, fixation vector, framework rigidity, and the ability to distribute occlusal loads across multiple anatomical support zones. The implant should not be conceived as a passive mesh lying on bone, but as a rigid, load-distributing skeletal framework stabilized by multivectorial fixation.

This concept also explains why modern CAD/CAM subperiosteal implants cannot be judged according to the same assumptions that applied to historical cast subperiosteal implants. Older devices were limited by inaccurate adaptation, absence of rigid fixation, and poor biomechanical planning, which contributed to mobility, epithelial invagination, infection, and progressive exposure [12,13]. By contrast, contemporary devices are planned from CBCT or CT datasets, prosthetically reverse-engineered from a wax-up or digital prosthetic plan, manufactured with additive or subtractive CAD/CAM techniques, and fixed to stable bone with osteosynthesis screws [14,113,122]. This digital workflow has shifted the biomechanical logic of subperiosteal implants from tissue-supported retention to rigid skeletal fixation.

However, this fixation-based model also implies that mechanical failure may occur when any component of the load-bearing chain is inadequate. Poor passive fit may generate micromotion and soft-tissue irritation; insufficient screw fixation may increase mobility; inadequate framework thickness may cause deformation; unfavorable abutment distribution may increase cantilever forces; and poor prosthetic control may overload screws or transmucosal components [19,20,21,54]. For this reason, modern subperiosteal implant planning should be understood as a combined surgical, prosthetic, and biomechanical procedure rather than as a purely implantological one.

### 5.2. Anchorage Zones

The choice of anchorage zones is one of the most important determinants of implant stability. In the maxilla, the most frequently described support areas include the nasomaxillary pillar, the zygomaticomaxillary buttress, the canine pillar, the palate, and, in more recent or complex designs, the pterygoid process, zygomatic arch root, and lateral skull base [25,45,48,115]. These regions are preferred because they are less affected by alveolar resorption than the edentulous ridge and provide thicker cortical bone for screw fixation. In maxillary full-arch and sectional rehabilitations, fixation to the nasomaxillary and zygomaticomaxillary buttresses has been repeatedly emphasized as a way to distribute occlusal loads away from the resorbed alveolar crest and toward more stable skeletal structures [19,33,53].

The palate may provide additional support in selected cases, particularly when palatal bone thickness is sufficient and when prosthetic or anatomical constraints require reinforcement. However, palatal screw access may be technically demanding, and dedicated instrumentation or modified screwdrivers may be required to achieve the intended screw vector [123]. More recently, pterygoid anchorage has been proposed as a posterior extension of maxillary subperiosteal implant fixation. In a case report, pterygoid bicortical screws were used to increase posterior support and potentially reduce anterior cantilever forces, although this remains an early concept requiring biomechanical validation and longer clinical follow-up [45].

In oncologic and post-ablative maxillary defects, conventional buttresses may be partially or completely absent. In such cases, the design must be adapted to the residual anatomy rather than transferred directly from severe atrophy protocols. Vosselman et al. emphasized that post-maxillectomy patients differ from atrophic edentulous patients because residual bone support is altered, soft tissues may be fragile or irradiated, and the framework may need to be thinner, less bulky, and validated by biomechanical assessment [124]. More recent series have extended anchorage to the pterygoid process, zygomatic arch root, and lateral skull base when traditional maxillary buttresses are insufficient, illustrating the evolution of PSSI from dental devices into craniofacial reconstructive platforms [43].

In the mandible, anchorage principles are different. The main fixation areas include the external oblique ridge, the lateral cortical plate, the basal mandibular bone, and regions that allow stable screw placement while avoiding the inferior alveolar nerve and mental foramen [34,85,118]. Posterior mandibular atrophy is particularly challenging because the residual bone height above the inferior alveolar canal may be insufficient for endosseous implants, while nerve lateralization or vertical augmentation may carry relevant morbidity. PSSI may therefore provide a graftless alternative, provided that the framework avoids superficial crossing over the mental nerve emergence and obtains fixation from stable cortical areas [34,70]. In this context, the relationship between the framework, mental foramen, and residual crest is critical, because an excessively superficial framework may increase the risk of mucosal thinning, dehiscence, and late exposure.

### 5.3. Framework Design

Framework design has emerged as a major determinant of biomechanical behavior. Several FEA studies have compared monoblock versus dual designs, one-piece versus two-piece configurations, I-shaped versus Y-shaped wings, different abutment distributions, posterior extensions, diagonal bars, pterygoid anchorage, and buttress-based fixation strategies [26,50,83,84,86,117]. Although these studies are heterogeneous, a consistent message emerges: the design should minimize bending moments, avoid unnecessary cantilevers, distribute loads across stable buttresses, and preserve sufficient rigidity without excessive bulk.

Dual or segmented designs may offer advantages in selected maxillary cases by reducing stress compared with monoblock configurations and by facilitating surgical handling, prosthetic passivity, and complication management [26]. This is consistent with clinical protocols in which bilateral maxillary subperiosteal implants are designed as two independent devices rather than as a single full-arch framework, in order to reduce cumulative fitting errors, avoid excessive palatal dissection, and allow isolated management if one side develops complications [25,71]. However, modularity must be balanced against mechanical continuity. In mandibular FEA, two-piece designs may increase framework stress compared with single-piece designs because of discontinuity at the abutment–framework connection, suggesting that modular designs should be adopted cautiously and tested under realistic loading conditions [86].

The shape of fixation wings also influences stress distribution. I-shaped designs appear favorable when anatomy permits, whereas Y-shaped or more complex extensions may be required in anatomically constrained cases but can increase displacement or stress depending on material and loading conditions [50]. Similarly, diagonal bars or alternative zygomatic extensions do not automatically improve biomechanics. In a total maxillectomy model, an alternative diagonal bar design did not provide a clear advantage over a conventional subperiosteal design and showed higher stress under oblique loading in nasomaxillary and zygomaticomaxillary regions [117]. These findings suggest that design modifications should be validated biomechanically rather than assumed to be beneficial simply because they increase framework extension.

Cantilever control is another recurring principle. Several studies indicate that posterior support and broader load distribution reduce stress concentration, whereas cantilevered prosthetic segments increase displacement and mechanical demand on screws and framework arms [19,21,50]. Clinically, this supports the use of posterior anchorage whenever feasible and explains the growing interest in pterygoid, zygomatic, or skull-base landing zones in complex maxillary defects [43,45].

The design of coupling elements and transmucosal posts is equally relevant. Long-term analysis of patient-specific subperiosteal implants with coupling elements showed that reduced distance between coupling elements, particularly less than 10 mm, and close proximity to natural teeth were associated with higher complication rates, probably because of biofilm accumulation, soft-tissue crowding, and reduced hygienic access [48]. Thus, abutment number and distribution should not be determined only by prosthetic support requirements, but also by soft-tissue thickness, access for hygiene, emergence profile, and long-term maintainability.

In hybrid rehabilitations combining subperiosteal and endosseous implants, the framework should remain structurally autonomous. The presence of endosseous implants should not justify simplification of the subperiosteal framework or reduction in fixation to stable buttresses. Rather, the hybrid concept should use endosseous implants where native bone is adequate and reserve subperiosteal implants for critically deficient regions, while maintaining independent mechanical stability of each support system [32,108]. This principle is important both biomechanically and medico-legally, because conventional endosseous implants remain better documented when sufficient bone is available, whereas subperiosteal implants should be used where their specific graftless advantage is justified.

### 5.4. Thickness, Screws, and Fatigue

Framework thickness, screw configuration, and fatigue resistance represent key parameters for the structural safety of custom-made subperiosteal implants. Reducing framework volume may improve soft-tissue coverage and decrease bulk, but excessive thinning can compromise mechanical resistance. FEA studies evaluating implant thickness suggest that 1 mm frameworks may exceed material yield limits under functional loading, whereas 1.5 mm may represent a more reasonable lower threshold in several models, depending on material, geometry, and loading assumptions [49]. This is clinically relevant because the desire to reduce exposure risk by minimizing framework volume must be balanced against the need to avoid deformation, fatigue, or fracture.

Screw diameter and distribution also influence stress patterns. Kundakcioglu and Gedik compared 1.5 mm and 2.0 mm fixation screws and found that 2.0 mm screws reduced stress on bone and implant components, although smaller screws may produce different displacement profiles [119]. These findings support the clinical tendency to use 2.0 mm screws for primary fixation when bone anatomy allows, with larger rescue screws when adequate torque is not achieved. However, screw diameter alone is insufficient; screw length, bicortical engagement, insertion vector, number of screws per arm, and anatomical distribution all contribute to stability.

Advanced FEA studies have also shown that simplified screw modelling may underestimate or misrepresent stress distribution. Castrillo et al. demonstrated that explicit modelling of screw-to-bone interaction and thread engagement can change displacement and stress results, especially in cortical bone regions adjacent to fixation screws [82]. This is particularly important because fixation screws, rather than the subperiosteal framework alone, frequently act as the main mechanical interface between the device and the patient’s skeleton. Future biomechanical studies should therefore avoid overly simplified boundary conditions and should include realistic modelling of cortical thickness, screw geometry, thread contact, and bone quality.

Fatigue testing is another area of increasing importance. While many FEA studies provide static stress analyses, clinical implants are exposed to millions of loading cycles, including oblique forces, parafunction, and prosthetic cantilever loads. Vanaclocha et al. combined iterative FEA optimization with mechanical testing of laser-powder bed fusion Ti6Al4V subperiosteal implant designs and reported survival under static and fatigue testing conditions, including cyclic loading for five million cycles [54]. This type of experimental validation is particularly valuable because it moves beyond theoretical stress mapping and begins to establish preclinical safety standards for future devices.

Topological optimization may also contribute to safer and less invasive designs. Carnicero et al. used topology optimization to reduce framework volume while maintaining mechanical performance, suggesting that additive manufacturing can be used not only for customization but also for genuine biomechanical optimization [20]. Similarly, realistic FEA studies indicate that adding posterior fixation, increasing the distribution of screws, and improving load pathways may reduce stress concentration more effectively than simply increasing framework bulk [21]. Overall, the available evidence suggests that thickness, screw configuration, and fatigue resistance should be treated as integrated design variables rather than isolated technical details.

### 5.5. Materials

Titanium and Ti6Al4V remain the reference materials for contemporary PSSI. Their widespread use reflects a combination of favorable mechanical strength, biocompatibility, additive manufacturing experience, sterilization protocols, and clinical familiarity from maxillofacial osteosynthesis and endosseous implantology [14,17,25,43]. Most clinical series and case reports of modern subperiosteal implants have used titanium alloys, particularly Ti6Al4V, making titanium the only material with a meaningful clinical evidence base in this field.

Alternative materials have been explored mainly through FEA, small clinical series, or early case reports. PEEK, PEKK, BioHPP, and carbon-fiber-reinforced PEEK are attractive because of their lower elastic modulus, radiolucency, and potential stress-absorbing behavior. Mounir et al. compared titanium and PEEK patient-specific subperiosteal implants in a small clinical cohort with short follow-up and found stable implants in both groups, although the sample was too small to establish material superiority [69]. El-Sawy et al. later reported a small case series of PEEK subperiosteal frameworks for fixed maxillary prostheses with stable short-term outcomes and no major complications at 12 months [52]. These studies indicate feasibility, but they do not yet provide sufficient evidence to replace titanium as the reference material.

Biomechanical studies offer a more nuanced picture. Altıparmak et al. reported that carbon-fiber-reinforced PEEK may produce lower stresses within the implant system while maintaining similar stress values in cortical and trabecular bone compared with titanium [121]. Baş et al. suggested that PEEK subperiosteal implants may behave favorably under traumatic loading in the severely atrophic mandible, with lower and more balanced stress values in some components than titanium designs [118]. However, other FEA studies have been less favorable toward polymeric frameworks in full-arch maxillary loading. El-Sawy et al. found that titanium frameworks transferred less stress to bone and screws and maintained better stability than modified PEEK or PEKK combinations under several loading scenarios, while polymeric materials could generate critical stresses in the cement layer, framework, or bone under anterior loading [80]. Similarly, Demir and Caglar reported that PEEK-composite prosthetic frameworks increased stress on bone, implant components, and prosthetic screws compared with more rigid frameworks, whereas CoCr and zirconia showed more favorable biomechanical behavior in their model [51].

These apparently conflicting results should not be interpreted as simple contradictions, but as evidence that material behavior depends strongly on anatomical site, loading scenario, prosthetic design, framework geometry, and the specific component being evaluated. A material that reduces stress within the framework may increase displacement or screw stress; a more rigid material may reduce deformation but concentrate stress elsewhere; and the ideal material for a mandibular trauma model may not be ideal for a maxillary full-arch prosthesis. Therefore, clinical extrapolation from FEA material studies should be cautious.

The same caution applies to CoCr and zirconia. In prosthetic superstructures, rigid materials such as CoCr or zirconia may reduce displacement and screw stress in certain models, but this does not necessarily mean that they are ideal for the subperiosteal implant framework itself [50,51]. The distinction between implant framework material, prosthetic framework material, veneering material, abutment connection, and screw material is essential. Some studies suggest that the framework material has a greater effect on stress distribution than the veneering material, and that prosthetic screws may represent a critical mechanical region, particularly in modular or full-arch rehabilitations [51,116].

At present, titanium/Ti6Al4V should therefore be regarded as the clinical reference standard for custom-made subperiosteal implants. PEEK, PEKK, BioHPP, carbon-fiber-reinforced PEEK, CoCr, and zirconia are promising in selected components or indications, but their use should be guided by anatomical context, prosthetic design, loading conditions, and long-term validation. The current literature supports material innovation, but not premature clinical generalization. Future studies should combine realistic FEA, fatigue testing, biological evaluation, and prospective clinical outcomes before alternative materials can be recommended beyond carefully selected cases.

## 6. Surface Biology and Osteogenic Potential

### 6.1. Biological Rationale for Modern Surface-Treated Subperiosteal Implants

Modern PSSI differ from historical cast frameworks not only in digital design and rigid fixation, but also in the possibility of engineering tissue-facing surfaces. Surface topography, post-processing, and selective finishing of bone-facing and transmucosal regions may influence both biological response and complication risk, although most evidence remains preclinical [12,13,14,25,33,61,113]. The biological question is therefore no longer limited to whether a metallic framework can be tolerated beneath the periosteum. Rather, contemporary research is beginning to ask whether additively manufactured titanium surfaces can promote a favorable hard-tissue response, whether bone apposition may occur on selected portions of the framework, and how the transmucosal interface can be optimized to reduce mucositis, recession and exposure. The available evidence remains heterogeneous and largely preclinical, but it supports the view that modern subperiosteal implants should be evaluated separately from legacy devices [87,91,93,94,125].

A key translational contribution was provided by Cohen et al., who developed an additively manufactured Ti6Al4V device with micro- and nano-textured surface features intended to stimulate osteogenic and angiogenic pathways. Their experimental work showed increased expression of osteogenic mediators in vitro and improved bone growth, bone-to-implant contact and pull-out strength in animal models, with preliminary clinical observations suggesting new bone formation and device stability [87]. Although this study does not directly establish clinical osseointegration of contemporary dental subperiosteal frameworks, it provides an important proof of concept: titanium devices positioned on bone surfaces can be engineered to elicit a more favorable biological response than simple mechanical contact alone.

### 6.2. Bone Apposition and the Unresolved Question of Osseointegration

The term “osseointegration” should be used with caution in the field of subperiosteal implants. In conventional endosseous implantology, osseointegration implies direct structural and functional bone-to-implant contact under load, ideally supported by histological or histomorphometric evidence. PSSI, however, have a different biological and mechanical environment. They are placed on the external cortical surface, stabilized by fixation screws and connected to the oral cavity through transmucosal abutments. Their primary stability is therefore fixation-based, not osseointegration-based.

Radiographic studies suggest that PSSI may show limited bone remodeling when the abutments are properly seated on basal bone. Van den Borre et al. reported minimal crestal remodeling after maxillary PSSI placement, without radiographically significant progressive atrophy around supporting regions at one year [95]. In sectional maxillary rehabilitations, minimal bone loss beneath the abutments was similarly observed when the crestal housing was prepared to allow the abutment to rest on stable basal bone rather than on residual alveolar crest [33]. These data support a key technical principle: progressive bone resorption beneath transmucosal components may be reduced when residual unstable alveolar bone is removed and the abutment is seated on more stable basal bone.

A preliminary observation by the present author group, adds a tentative element to this discussion. In a three-case report, surgical re-entry in selected symptomatic cases allowed direct intraoperative observation of bone coverage or apposition on portions of the framework [125]. Because this is a small, non-primary report from our own group and lacks histological confirmation, it should be regarded as hypothesis-generating only and not as load-bearing evidence of a biological phenomenon. This observation is particularly relevant because most previous evidence of bone response around subperiosteal implants relied on radiographic inference, whereas re-entry provides direct clinical documentation of the implant–bone interface. At the same time, the interpretation must remain conservative. Without histological sampling and histomorphometric quantification, such findings are best described as clinical evidence of bone apposition or bone coverage, rather than definitive proof of osseointegration.

This distinction is not merely semantic. Bone apposition may contribute to secondary biological stabilization of the framework and may help explain why some devices remain clinically stable beyond their initial screw fixation. However, it does not eliminate the central role of passive fit, rigid fixation, screw distribution, framework design and controlled loading. In other words, modern subperiosteal implants may benefit from favorable bone response over time, but their clinical reliability cannot be assumed to depend on osseointegration in the same way as conventional endosseous fixtures.

From a methodological standpoint, Babuska et al. emphasized that robust evaluation of osseointegration requires adequate experimental models, histology, histomorphometry and quantification of bone–implant contact [88]. This remains a major gap in the subperiosteal implant literature. Future studies should therefore combine clinical re-entry observations, high-resolution radiological follow-up, retrieval analysis when available and histological confirmation in selected cases.

### 6.3. Osteogenic Response to Titanium Surface Treatments

Several in vitro studies have investigated how different Ti6Al4V surface treatments influence osteoblast-like cells, osteogenic markers and extracellular matrix maturation. Roy et al. evaluated titanium surfaces with different topographies and treatments, reporting that the tested surfaces did not impair cell viability and were able to stimulate the expression of osteogenic and adhesion-related markers, including RUNX2, osteocalcin, osterix, N-cadherin, β-catenin and osteoprotegerin [92]. These findings suggest that modern titanium surfaces may support osteoblast activity and matrix-related signaling.

Campagna et al. compared several Ti6Al4V surface conditions, including control, electroerosion-polished, etched and sandblasted, Al Ti Color and color-anodized surfaces. Their results suggested that etched and sandblasted surfaces promoted early proliferation, whereas the Al Ti Color treatment appeared to generate a more favorable osteogenic differentiation, mineralization and adhesion-related gene-expression profile [93]. This distinction is important because not all biologically active surfaces act through the same mechanism. Some may primarily support early cell proliferation, while others may favor later differentiation and mineralized matrix deposition.

Schiavoni et al. further supported this concept by showing that ATcs-treated Ti6Al4V surfaces promoted markers related to osteogenic maturation, including DMP1 expression, organized collagen deposition and calcium–phosphate mineralized structures [94]. Together, these studies provide a biological rationale for the use of treated titanium surfaces on bone-facing regions of modern subperiosteal implants. However, their interpretation must remain cautious. Most data derive from in vitro models, often using osteoblast-like cell lines, which cannot fully reproduce the subperiosteal environment, periosteal vascularity, cortical bone remodeling, bacterial contamination, mechanical micromotion and prosthetic loading.

The clinical observations of bone apposition reported by our own group should therefore be read with caution: at most they hint at a possible clinical counterpart to the osteogenic potential seen in laboratory studies [125], but they cannot bridge the gap between preclinical surface biology and clinical behaviour in the absence of histological confirmation.

They suggest that the osteogenic potential observed in laboratory studies may have a clinical counterpart in selected cases. Nevertheless, these findings should be interpreted as hypothesis-generating rather than definitive, and they reinforce the need for future studies specifically designed to correlate surface treatment, implant location, time in function, bone apposition and clinical stability.

### 6.4. Soft-Tissue Interface and Transmucosal Surface Considerations

Although bone response is increasingly recognized as relevant, the clinical success of subperiosteal implants may depend even more critically on the transmucosal interface. Contemporary devices include extraosseous frameworks and abutments that cross the mucosa, creating potential plaque-retentive areas and pathways for bacterial contamination of the substructure. Therefore, the optimal surface is not necessarily the same for all portions of the implant.

Roy et al. investigated the response of gingival fibroblasts to different titanium surfaces and found no relevant cytotoxic or pro-inflammatory effect. Several surfaces stimulated extracellular matrix-related genes, and sandblasted/acid-etched surfaces appeared to promote fibroblast proliferation and migration [91]. These findings are relevant because fibroblast adhesion and extracellular matrix deposition may contribute to a more stable soft-tissue seal around transmucosal components. However, this potential benefit must be balanced against the risk of bacterial adhesion.

Ardhani et al. reviewed the relationship between titanium surface topography and Porphyromonas gingivalis adhesion, suggesting that smoother surfaces may reduce bacterial adhesion and that lower roughness values may be preferable for exposed or transmucosal components [90]. This is particularly important in subperiosteal implants, where exposure or mucosal recession can directly expose portions of the framework to the oral environment.

Clinical studies reinforce the importance of this balance. Van den Borre et al. reported recession or framework exposure in 65% of patients after bilateral maxillary PSSI placement, with thin mucosal biotype and mucositis identified as significant risk factors; smoking showed a strong, although not statistically significant, association with recession [46,126]. Similarly, systematic reviews consistently identify dehiscence, exposure, mucositis and soft-tissue inflammation as the most frequent complications of modern subperiosteal implants, despite generally high survival rates [28,55,56].

These findings suggest that surface biology should be integrated into a broader soft-tissue strategy. A rough or osteogenic surface may be advantageous in bone-contacting areas, but transmucosal and potentially exposed components should probably be polished, smooth and highly cleanable. This concept is consistent with clinical protocols describing polished transmucosal portions, microtextured bone-facing portions and meticulous soft-tissue thickening to reduce exposure risk [33,40,71].

### 6.5. Hybrid Surface Concepts: Bone-Facing Versus Transmucosal Regions

The available evidence supports the development of hybrid surface concepts for PSSI. Bone-facing regions may benefit from surface treatments intended to enhance osteogenic response, such as sandblasting, acid etching, anodization, Al Ti Color treatment or other micro-/nano-topographical modifications. In contrast, transmucosal regions should probably prioritize soft-tissue compatibility, plaque control and ease of professional maintenance.

This anatomical differentiation is particularly relevant because the same implant may simultaneously interact with bone, periosteum, connective tissue, oral mucosa and bacterial biofilm. A uniform surface treatment across the whole device may therefore be biologically suboptimal. The bone-contacting frame should aim to promote bone stability or apposition, whereas the transmucosal components should minimize plaque retention and chronic inflammation.

The long-term design-related study by Pott et al. indirectly supports this principle. The authors reported that soft-tissue and inflammatory complications increased over time and were associated with design-related factors, including short distances between coupling elements and proximity to natural teeth. They recommended adequate spacing between coupling elements, avoidance of excessive proximity to teeth, high-gloss transmucosal surfaces and structured professional maintenance [48]. These recommendations are consistent with the idea that biological success depends not only on surface chemistry but also on cleanability, prosthetic access and mucosal stability.

In this perspective, the clinical documentation of bone apposition reported by Vaira et al. should not lead to overemphasis on the bone-facing interface alone [125]. Even if bone apposition can occur on selected parts of the framework, complications may still arise through the transmucosal pathway. Therefore, future device optimization should pursue a dual objective: enhancing bone response where the implant contacts bone, while reducing biofilm accumulation and soft-tissue inflammation where the device communicates with the oral cavity.

## 7. Clinical Outcomes and Complications

### 7.1. Survival, Success and Interpretation of Clinical Endpoints

**Figure 2:**
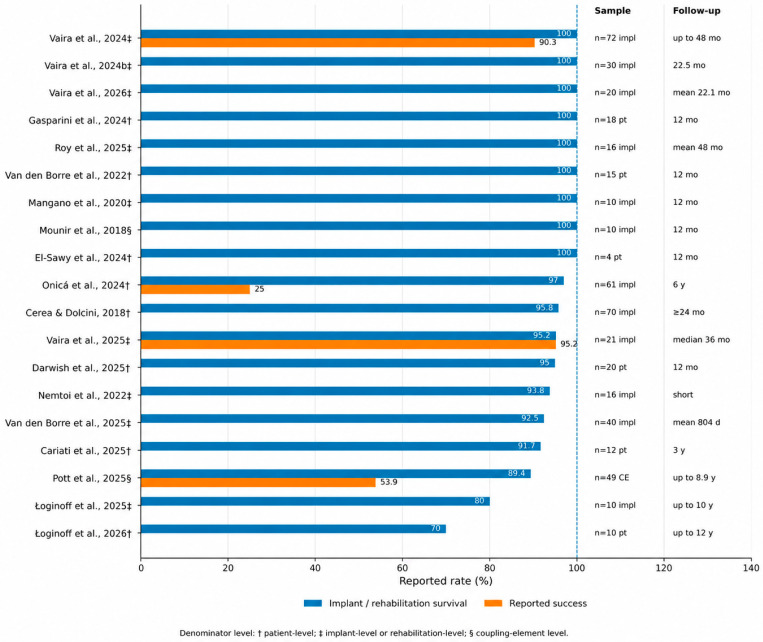
Reported implant/rehabilitation survival and, where separately reported, success in clinical studies of contemporary custom-made subperiosteal implants [17,18,22,24,25,31,32,33,34,35,48,52,69,96,97,104,105,106,107].

The available clinical evidence on contemporary PSSI suggests generally favorable short- and mid-term survival, particularly when compared with the historical experience of cast subperiosteal frameworks [Figure 2]. As shown in Figure 2, however, survival and success do not coincide: cohorts with high implant survival may report substantially lower case-level success (for example, Onică et al. reported ≈97% of implants in situ but only 25% of successful cases), and reporting levels differ across studies, so survival figures should not be read as interchangeable across denominators. However, interpretation of survival data requires caution because included studies differ substantially in clinical indication, jaw site, prosthetic design, loading protocol, follow-up duration and definition of success.

Across reviews and primary clinical series, reported survival of contemporary PSSI is generally high in the short and mid term, especially for digitally planned and additively manufactured devices [17,18,25,27,28,29,34,56,105]. However, the interpretation of these figures is limited by heterogeneous indications, variable follow-up, overlapping cohorts, and inconsistent definitions of success. Longer-term series and design-related analyses show that survival and success may diverge over time, particularly when recurrent infection, soft-tissue inflammation, progressive exposure, or coupling-element complications occur [48,97,105,107]. Therefore, survival should be interpreted as a minimum endpoint rather than as proof of durable biological success.

A further difficulty is that survival does not necessarily correspond to success. Survival usually indicates that the device remains in situ and supports a functional prosthesis. Success, however, should also consider absence of mobility, absence of persistent infection, acceptable soft-tissue conditions, absence of progressive framework exposure, stable fixation screws, prosthetic function, patient satisfaction and maintenance burden. Several authors have therefore emphasized the need for procedure-specific success criteria for subperiosteal implants, rather than the direct transfer of criteria developed for endosseous implants [32,33,55] because their fixation-based biology and transmucosal interface (Section 5.1) entail risks that differ from those of conventional endosseous fixtures.

### 7.2. Biological Complications

Biological complications represent the most clinically relevant adverse events reported in contemporary subperiosteal implant literature. They include mucosal dehiscence, framework exposure, mucositis, soft-tissue recession, infection, suppuration, delayed inflammatory episodes, pain, edema, sinus-related events and, in selected long-term cases, progressive bone resorption around the framework or abutments.

Soft-tissue dehiscence and framework exposure are the most frequently reported biological complications. Anitua et al. found partial implant exposure in 25.6% of patients and persistent soft-tissue infection or inflammation in 5.3% [28]. Al-Nawas and Bär reported partial exposure in 37% of cases across the included digital workflow literature [56]. El-Sawy and Hegazy reported biological complications in 11.5%, mainly dehiscence and exposure [29]. Ruiz-Rincón et al., focusing specifically on CAD/CAM subperiosteal implants supporting fixed partial restorations, reported a biological complication rate of 4.13%, including wound dehiscence, bone loss and implant exposure [55].

The incidence of exposure varies widely across clinical series, likely reflecting differences in implant design, soft-tissue phenotype, surgical technique, prosthetic protocol and follow-up duration. In the multicenter full-arch maxillary study by Vaira et al., limited asymptomatic framework exposure occurred in 9.7% of implants, without implant loss and without significant bone resorption beneath abutments [25]. In the sectional maxillary series, no framework exposure was observed over a median follow-up of 36 months, possibly reflecting specific technical measures such as palatal incision placement, deep abutment seating in crestal slots, fixation to maxillary buttresses and selective soft-tissue thickening using buccal fat pad or membranes [33]. Conversely, Dimitroulis et al. reported a higher complication burden in their preliminary experience with a new generation of CAD/CAM subperiosteal implants, with framework exposure representing the main adverse event; several cases were salvaged, but the primary success rate was only 66.7%, increasing to 85.7% after successful management of complications [23]. The study by Van den Borre et al. is particularly important because it specifically evaluated soft-tissue response and risk drivers after bilateral maxillary PSSI placement. In 40 patients with severe maxillary atrophy, recession or framework exposure was observed in 26 patients, corresponding to 65% of the cohort. Thin mucosal biotype and the presence of mucositis were significantly associated with recession, while smoking showed a strong but non-significant association, with an odds ratio of 6.88 [46]. These findings indicate that successful subperiosteal implant rehabilitation depends not only on mechanical stability but also on mucosal phenotype, biofilm control, patient compliance and prevention of mucositis.

Mandibular PSSI data show a somewhat different but still relevant soft-tissue profile. Van den Borre et al. reported mucosal recession around 13 of 40 mandibular implants, corresponding to 32.5%, although patients did not regard this as a functional or aesthetic concern [106]. In the long-term design-related study by Pott et al., soft-tissue inflammation increased over time and was associated with biofilm accumulation around coupling elements, short distances between coupling elements and proximity to natural teeth [48]. These observations suggest that soft-tissue complications may be partly device-related and partly hygiene-related, and that implant design should anticipate professional and home-care accessibility.

Infection is less frequent than exposure but more clinically consequential. Cerea and Dolcini reported three implant losses due to recurrent infections [17]. Nemtoi et al. reported one implant loss related to poor fit and recurrent infections [18]. Łoginoff et al. reported maxillary and mandibular late removals related to recurrent infection, poor fit, suppuration or granulation tissue [97,105]. Surana et al. described infection and removal of one patient-specific subperiosteal implant in a post-COVID mucormycosis rehabilitation, followed by prosthetic salvage using an overdenture supported by the remaining framework and bar system [76]. These cases highlight the need to consider not only implant placement but also implant removability, modularity and salvage options during the planning phase.

Wound dehiscence is another important event because it may represent the first step toward contamination, exposure, infection and implant loss. Mounir et al. reported one dehiscence in the titanium group in a small clinical comparison of titanium and PEEK patient-specific subperiosteal implants [69]. Vaira et al. reported one implant failure in a heavy smoker after wound dehiscence and infection in the sectional posterior maxillary series [33]. Cariati et al. reported complications in four of twelve patients treated with virtual-planned maxillary subperiosteal implants, including two exposures and one failure related to insufficient bone reshaping that required revision [96]. These observations reinforce the importance of incision design, tension-free closure, keratinized tissue repositioning, soft-tissue thickness and smoking control.

Delayed non-suppurative edema has emerged as a distinct clinical pattern in some modern subperiosteal implant cohorts. This phenomenon, particularly described in maxillary cases, may present as recurrent swelling without radiological evidence of infection or implant loosening and may respond to conservative anti-inflammatory treatment or selective management of fixation screws [102]. Although the pathophysiology remains unclear, it may reflect low-grade inflammatory reactions around osteosynthesis hardware, local soft-tissue strain or immune response to micromotion or screw-related irritation. Future studies should distinguish this entity from infectious swelling, because management strategies may differ substantially.

Pain and edema are frequently reported in the immediate postoperative period, but they should not always be classified as complications. Several studies describe edema and discomfort as expected postoperative sequelae, usually self-limiting and responsive to standard analgesic and anti-inflammatory protocols [25,31,56,111]. However, chronic pain, persistent swelling, suppuration or pain associated with mobility should be interpreted as clinically relevant adverse events requiring further investigation.

Sinus-related complications appear less frequent than in zygomatic implant literature, but they remain relevant in maxillary designs involving sinus walls, zygomatic buttresses, pterygoid extensions or post-oncologic defects. Zielinski et al. reported that sinus-related complications were more frequent in zygomatic implants than in subperiosteal implants in a comparative 5-year study of patients with severe maxillary atrophy, whereas peri-implantitis was reported in 5.6% of subperiosteal implant cases [127]. Technical studies emphasize preservation or repair of the Schneiderian membrane during abutment housing preparation and careful planning of fixation screws away from thin sinus walls [25,33].

### 7.3. Mechanical and Prosthetic Complications

Mechanical and prosthetic complications appear less frequent than biological complications in most contemporary studies, but they are clinically important because modern subperiosteal implants are mechanically complex structures. Reported complications include prosthetic screw loosening, fixation screw loosening, crown recementation, provisional prosthesis fracture, implant instability, abutment-related problems, framework stress and, rarely, implant mobility.

Framework fracture appears uncommon in the available clinical literature. Most modern titanium frameworks are designed using CAD/CAM workflows, manufactured in Ti6Al4V or other high-performance materials, and fixed with multiple osteosynthesis screws. Clinical series generally do not report structural framework fractures in the short or mid term [17,25,33,35]. Nevertheless, finite element and mechanical validation studies show that framework thickness, material selection, cantilever length, fixation screw distribution and connection design can substantially influence stress concentration and fatigue risk [19,49,54,58].

Prosthetic complications are more frequently described. Mangano et al. reported provisional restoration fractures during the temporization phase [31]. Ruiz-Rincón et al. reported a mechanical complication rate of 7.44% in fixed partial restorations, including provisional restoration fracture, implant instability and crown recementation [55]. Roy et al. reported two cases requiring recementation of crowns after three years in patients treated with subperiosteal implants for lateral incisor agenesis [35]. These events are generally manageable, but they highlight the importance of provisional design, occlusal control, avoidance of lateral overload and periodic prosthetic maintenance.

Screw-related complications deserve particular attention. In clinical studies, fixation screw loosening is relatively uncommon when passive fit and adequate fixation are achieved, but screw stress is repeatedly identified as a critical biomechanical variable. Vaira et al. reported no fixation screw loosening in posterior mandibular and sectional maxillary cohorts [33,34]. However, other studies have reported implant instability or mobility in selected cases, often associated with infection, poor fit, insufficient fixation or adverse loading [18,106,111]. Vörös et al. showed in vitro that tightening torque is critical for connection stability, with 30 Ncm maintaining stability more effectively than 15 Ncm after cyclic loading of M1.8 connection screws [116]. Although this study concerns connection screws rather than bone fixation screws, it underscores that modular subperiosteal systems may be vulnerable to screw loosening if torque protocols are inadequate.

Finite element studies provide a mechanistic explanation for these observations. Demir and Caglar found that prosthetic screws may represent the area with the highest stress in full-arch subperiosteal rehabilitations, particularly when less rigid prosthetic framework materials such as PEEK-composite are used [51]. Castrillo et al. showed that modelling the screw-to-bone interface can substantially alter predicted displacements and cortical stress, demonstrating that screw geometry and cortical engagement are not negligible details in biomechanical simulations [82]. Pellegrino et al. similarly emphasized that screw number, posterior fixation and load distribution influence stress concentrations in realistic atrophic jaw models [21].

Implant mobility is one of the clearest indicators of failure or impending failure. Historical implants frequently failed through mobility, infection and progressive tissue breakdown. In modern series, mobility is less frequent but still reported. Van den Borre et al. found one mandibular PSSI with mobility greater than 1 mm, two implants removed for persistent infection and suppuration, and another scheduled for removal [106]. In the maxillary soft-tissue study, one patient showed mobility greater than 1 mm of bilateral PSSI at the time of evaluation [46]. These findings suggest that mobility should remain a major failure criterion, especially when associated with suppuration, pain or progressive radiological changes.

### 7.4. Exposure Is Not Always Failure

A central conceptual point in the evaluation of contemporary subperiosteal implants is that framework exposure should not automatically be equated with implant failure. The clinical meaning of exposure depends on its extent, location, symptoms, inflammatory status, progression and effect on prosthetic function.

Small, stable, asymptomatic exposures confined to vertical transmucosal components or limited portions of the framework may behave as minor sequelae rather than true failures, particularly if there is no suppuration, pain, mobility, progressive enlargement or prosthetic impairment [47,102]. This is consistent with clinical studies in which limited exposure did not necessarily lead to implant removal or loss of function [25,55,106]. In contrast, progressive exposure associated with mucositis, infection, suppuration, pain, recurrent swelling or mobility should be considered a clinically significant complication and may require surgical revision or implant removal.

The need for standardized severity-based reporting of framework exposure has therefore become increasingly evident. At present, no universally accepted or validated exposure classification specific to PSSI is available. Previous work from the present group described limited, asymptomatic exposure separately from inflammatory, progressive, or mobility-associated exposure [25,102]; but this should be interpreted as an informal descriptive distinction rather than as a validated classification system. For this reason, the present review did not use exposure classes for outcome extraction or tabular reporting. Instead, when details were available, exposure was described narratively according to extent, symptoms, inflammatory status, progression, prosthetic impact, and association with mobility or implant removal. This distinction is important for both clinical management and scientific reporting. Without standardized reporting, studies that report “exposure” may group together biologically different events, ranging from minor soft-tissue recession to true implant failure.

This concept also affects the definition of success. A device may survive and remain functional despite minor exposure, but it may not satisfy strict success criteria if exposure is progressive, inflamed or requires repeated interventions. Conversely, classifying every small asymptomatic exposure as failure may underestimate the clinical utility of these devices in patients with limited alternatives. The most balanced approach is therefore to report survival, functional success, biological complications and exposure grade separately.

### 7.5. Patient-Related and Site-Related Risk Factors

Several patient-related and site-related factors appear to influence the risk of complications, although the available evidence remains insufficient for definitive risk prediction. Thin mucosal biotype, mucositis and smoking are among the most frequently implicated variables. Van den Borre et al. identified thin biotype and mucositis as significant risk factors for recession after maxillary PSSI implantation, while smoking showed a strong but non-significant association with recession [46]. Vaira et al. reported the only implant loss in their sectional maxillary series in a heavy smoker who developed wound dehiscence, infection and implant mobility [33]. Mommaerts emphasized caution or exclusion in patients with poor compliance, active smoking, uncontrolled diabetes, immunocompromised status or previous infectious complications, particularly when long-term maintenance cannot be guaranteed [47].

Bruxism and parafunction may also be relevant. Darwish et al. reported one failure in the milled group of their randomized clinical trial, attributed to bruxism [104]. De Moor et al. predicted higher fatigue risk in cases of extreme atrophy, bruxism and maximal occlusal forces [19]. These findings support careful occlusal planning, avoidance of cantilever overload, protective night guards in selected patients and cautious indication in severe parafunction.

Site-related risk factors include posterior maxillary atrophy, thin mucosa, high smile line, proximity to natural teeth, limited prosthetic hygiene access, irradiated tissues and post-oncologic soft-tissue deficiency. In the long-term analysis by Pott et al., coupling elements placed closer than 10 mm and coupling elements close to natural teeth showed more complications, probably because of increased biofilm retention and periodontal contamination [48]. In oncologic or post-resection cases, the risk profile may be further influenced by radiotherapy, flap bulk, scar tissue, limited vascularity and reduced hygiene accessibility [37,38,41,57,101].

### 7.6. Comparison with Conventional Graftless and Reconstructive Alternatives

The complication profile of subperiosteal implants differs from that of conventional endosseous implants, zygomatic implants and bone augmentation procedures. While endosseous implants primarily fail through osseointegration failure, peri-implantitis, marginal bone loss or prosthetic overload, subperiosteal implants are more strongly affected by soft-tissue stability, framework exposure, mucositis, fixation mechanics and biofilm access to the substructure.

Compared with zygomatic implants, subperiosteal implants may avoid some sinus-related or trajectory-related complications, but they introduce different risks related to the transmucosal interface and broad extraosseous frameworks. Zielinski et al. reported comparable 5-year survival between zygomatic and subperiosteal implants in severe maxillary atrophy, with more sinus-related complications in the zygomatic group and peri-implantitis reported in the subperiosteal group [127]. Sudhir et al. similarly suggested high short-term survival for both zygomatic and subperiosteal solutions, but with different dominant complications: sinusitis for zygomatic implants and soft-tissue dehiscence for subperiosteal implants [60].

Compared with guided bone regeneration or reconstructive grafting, PSSI may reduce treatment time, morbidity and the need for staged surgery, but they are not free from maintenance demands or biological risk. The decision should therefore be individualized, considering residual bone, patient age, comorbidities, willingness to undergo grafting, prosthetic expectations, hygiene capability and long-term follow-up feasibility.

## 8. Prosthetic Protocols and Maintenance

### 8.1. Loading Protocols and Prosthetic Timing

The prosthetic phase represents a key determinant of the clinical behavior of contemporary custom-made subperiosteal implants. Unlike historical subperiosteal frameworks, which were frequently used in delayed or staged rehabilitative pathways, modern CAD/CAM and additively manufactured systems are usually planned according to a prosthetically driven digital workflow, in which the position of the transmucosal components is defined from the diagnostic wax-up and final restorative objectives [14,33,113,128]. This reverse-planning approach has progressively shifted subperiosteal implantology from a purely surgical rescue technique to an integrated implant-prosthetic strategy.

Immediate or early loading has been reported in several contemporary clinical series, particularly in full-arch and segmental rehabilitations of severely atrophic jaws [17,25,31,33,69,129]. As detailed in Section 5.1, primary stability here derives from passive fit, rigid screw fixation and load distribution across stable buttresses rather than from endosseous osseointegration, and the loading strategy should be interpreted accordingly.

In full-arch maxillary rehabilitations, immediate loading with a screw-retained provisional prosthesis has been commonly described, with definitive prosthetic delivery after soft tissue maturation, usually several months after surgery [25,96,129]. Similar principles have been applied in posterior mandibular and sectional maxillary rehabilitations, where provisional prostheses were delivered immediately or within the first days or weeks after surgery, followed by definitive restorations after tissue conditioning [25,31,33]. However, delayed or conventional loading has also been used, particularly in earlier DMLS series or in protocols where soft tissue healing and adaptation were prioritized before functional loading [17,97,105].

The available evidence does not currently allow determination of whether immediate, early, or delayed loading is superior. Rather, loading should be individualized according to primary stability, framework fit, screw fixation, prosthetic passivity, occlusal risk, soft tissue quality, and patient-related factors such as bruxism, smoking, diabetes, previous radiotherapy, and compliance. In high-risk cases, particularly when tissue coverage is fragile or the prosthetic design is complex, delayed loading or a more protective provisional phase may be preferable.

### 8.2. Provisional and Definitive Prostheses

The provisional prosthesis should not be regarded only as a temporary restoration, but as a functional and biological conditioning device. It allows early restoration of aesthetics, phonetics, and mastication, but it also influences mucosal adaptation, plaque control, occlusal load distribution, and patient learning during the initial healing phase. For this reason, provisional prostheses should be designed with reduced occlusal overload, limited cantilever extension, passive fit, and adequate subprosthetic space for hygiene.

This is particularly relevant for subperiosteal implants because the soft tissue interface is more exposed to the oral environment than in conventional endosseous implants. The provisional prosthesis should allow the patient and clinician to access the transmucosal components and subprosthetic areas during the biologically vulnerable early period. In the maintenance-oriented framework proposed by Dessì and Vaira, the provisional phase is specifically considered an opportunity to leave adequate space between the prosthesis and the underlying tissues, facilitating hygiene, tissue monitoring, and patient adaptation before definitive prosthetic delivery [130].

The definitive prosthesis should preserve these hygiene principles while improving function, aesthetics, phonetics, and long-term mechanical resistance. Excessively convex or overcontoured designs may compromise home care and increase plaque retention under the prosthesis, particularly around transmucosal abutments. Conversely, excessive tissue contact may create pressure areas, reduce cleanability, and contribute to mucosal inflammation or dehiscence. Therefore, prosthetic design should aim to balance aesthetics and phonetics with biological maintainability.

Several materials have been reported for definitive restorations, including titanium bars with composite or acrylic resin, zirconia-based frameworks, milled titanium structures, and PEEK-based solutions [33,52,129]. Current evidence does not support a single ideal restorative material. Material selection should instead be based on prosthetic span, occlusal load, available restorative space, repairability, weight, hygiene accessibility, and risk of mechanical complications. In patients with parafunctional habits or high occlusal load, occlusal protection and careful control of cantilevers are especially important.

### 8.3. Fixed Versus Removable Prosthetic Solutions

Most contemporary reports describe fixed implant-supported rehabilitations, especially in patients seeking graftless restoration of severe atrophy with reduced treatment time [17,25,33,37,69]. Fixed solutions are often preferred by patients because they provide greater comfort, psychological acceptance, and perception of functional rehabilitation. In single-tooth or sectional indications, fixed restorations are also more consistent with conventional prosthodontic expectations.

However, fixed prostheses are not necessarily ideal in all contexts. In post-oncologic defects, flap reconstructions, obturator-supported rehabilitations, or cases with bulky soft tissue, removable or hybrid prosthetic designs may offer important advantages in terms of inspection, hygiene, and maintenance. Machine and Nadjmi described a palateless overdenture supported by a custom subperiosteal framework in a post-hemimaxillectomy patient, choosing a removable definitive solution to facilitate hygiene under the flap and improve long-term maintainability [101]. Similarly, obturator-retained approaches have been reported for post-ablative maxillary defects, where the prosthesis must restore separation between oral and sinonasal cavities as well as dental function [42,73].

Other authors have also emphasized the potential value of bar-supported removable overdentures in complex patient-specific rehabilitations, particularly when hygiene access, soft tissue protection, and long-term inspection are priorities [15,48]. Removable designs may reduce the burden of cleaning for patients with limited dexterity, irradiated tissues, bulky flaps, or complex subprosthetic anatomy. They may also facilitate professional debridement and early detection of mucosal inflammation or framework exposure.

Therefore, the choice between fixed and removable prostheses should not be framed as a hierarchy in which fixed is always superior. Rather, it should be based on indication, anatomy, patient expectations, hygiene capacity, soft tissue risk, and prosthetic accessibility. Fixed prostheses may be appropriate for stable atrophic jaws with favorable hygiene access, whereas removable or retrievable designs may be preferable in oncologic, reconstructive, geriatric, or highly complex cases.

### 8.4. Prosthetic Complications and Mechanical Surveillance

Mechanical and prosthetic complications reported in contemporary subperiosteal implant studies include provisional prosthesis fracture, crown recementation, prosthetic screw loosening, abutment-related complications, framework instability, and, less commonly, implant failure requiring removal [23,31,32,35,55]. Framework fracture appears uncommon in modern titanium devices, but the available follow-up remains limited and does not exclude fatigue-related complications over longer periods.

The prosthetic screw system may represent a mechanically vulnerable area. Several finite element studies have shown stress concentration around prosthetic connections, abutments, fixation screws, and coupling elements, particularly in the presence of cantilevers, unfavorable materials, oblique loading, or modular/two-piece designs [19,50,51,82,86]. In vitro data on screw tightening also suggest that inadequate torque may contribute to screw loosening under cyclic loading, reinforcing the need for manufacturer-specific torque protocols and periodic prosthetic assessment [116].

Clinical surveillance should therefore include occlusal evaluation, assessment of prosthetic passivity, inspection of screw-access channels, evaluation of prosthetic wear or fracture, and patient questioning regarding instability, noise, discomfort, or changes in mastication. In patients with bruxism or high occlusal load, occlusal splints may be considered, particularly during the provisional phase and after delivery of the definitive prosthesis [59].

### 8.5. Patient-Reported Outcomes and Quality of Life

Patient-reported outcomes are becoming increasingly important in the evaluation of contemporary patient-specific subperiosteal implants. In complex atrophic, congenital, post-oncologic, or salvage patients, implant survival alone may not adequately capture the clinical value of treatment. Improvements in oral function, speech, swallowing, comfort, esthetics, self-confidence, hygiene access, prosthetic manageability, and overall quality of life may be as relevant as the persistence of the implant in situ. Recent studies have incorporated patient satisfaction scales and OHIP-14 assessment, showing improvements in oral-health-related quality of life after rehabilitation in severely atrophic jaws [22,126]. Similarly, reconstructive and obturator-supported applications suggest that patient-specific frameworks may provide meaningful functional benefits in patients with limited conventional alternatives.

Future studies should therefore move beyond implant survival as the primary endpoint, incorporating patient-reported outcomes alongside the procedure-specific success criteria proposed in Section 7.1; as noted there, a surviving implant does not necessarily represent a successful rehabilitation.

### 8.6. Maintenance, Monitoring, and Management of Exposure

Long-term maintenance remains one of the least standardized aspects of custom-made PSSI rehabilitation. Most studies report survival and complications, whereas detailed hygiene protocols and recall strategies are rarely described. In the absence of dedicated comparative evidence, this section draws substantially on a narrative review by members of the present author group [130], which should be read as an expert proposal rather than as a validated protocol. Because PSSI are extraosseous frameworks with multiple transmucosal components, conventional peri-implant parameters such as probing depth or marginal crestal bone loss cannot be directly transferred to this setting. Maintenance should therefore rely on structured descriptive monitoring rather than rigid diagnostic thresholds.

At each follow-up visit, clinicians should document soft tissue condition, erythema, edema, bleeding tendency, suppuration, mucosal recession, framework exposure, plaque accumulation, prosthetic cleanability, pain, recurrent swelling, and patient-reported symptoms. Standardized intraoral photographs may be useful for comparing soft tissue changes and exposure progression over time. This descriptive approach has been proposed as a practical strategy until validated diagnostic criteria for subperiosteal implants become available [130].

Because no validated maintenance protocol exists, clinical management must currently be extrapolated cautiously from supportive peri-implant therapy, full-arch implant maintenance, zygomatic implant rehabilitation, and reconstructive surgery. The main objective is to reduce biofilm accumulation around transmucosal components, prosthetic margins, exposed surfaces, and subprosthetic spaces while avoiding trauma to fragile soft tissues. Home care should be simple, reproducible, and adapted to the patient’s anatomy and manual dexterity. Soft-bristle toothbrushes, interdental brushes, and selected single-tuft brushes may be used according to accessibility. Oral irrigators may be considered in selected patients, especially under full-arch prostheses or in wide subprosthetic spaces, but they should not replace mechanical plaque control. The key principle is not the universal prescription of a single device, but chairside demonstration and periodic reassessment of whether the patient can effectively clean the relevant areas.

Professional maintenance should include targeted biofilm disruption around transmucosal components, prosthetic margins, exposed implant areas, and subprosthetic spaces. Low-abrasive air-polishing powders, such as glycine or erythritol, and implant-safe ultrasonic tips may be considered when access and tissue conditions permit. Periodic prosthesis removal may be useful in selected cases, particularly when fixed prostheses limit inspection or debridement, but it should not be considered mandatory in all patients. In well-designed rehabilitations with adequate access and stable soft tissues, maintenance may often be performed without routine prosthesis disassembly. Conversely, prosthesis removal may be indicated in patients with recurrent inflammation, poor hygiene access, complex oncologic defects, unexplained symptoms, or progressive exposure. These principles are consistent with the proposed maintenance framework for PSSI, which emphasizes individualized professional hygiene according to prosthetic complexity, tissue condition, implant exposure, and patient-specific risk factors [130].

Recall intervals should also be individualized rather than fixed. An author-group-derived consensus report has suggested maintenance visits approximately every 5–6 months for customized subperiosteal implant restorations, with shorter intervals in patients with relevant risk indicators or non-hygienic prosthetic designs [59]; Because this source was co-authored by members of the present review group, this recommendation is interpreted here as concordant expert opinion rather than as independent confirmation. In clinically stable, low-risk patients with good hygiene access and no exposure progression, a 6-month recall may be reasonable. In patients with active mucositis, recurrent swelling, plaque accumulation, framework exposure, previous radiotherapy, oncologic defects, poor manual dexterity, smoking, diabetes, xerostomia, history of periodontitis, or limited prosthetic cleanability, 3–4-month intervals or shorter individualized recalls may be more appropriate. The interval should be reassessed at each visit according to clinical stability, tissue response, hygiene performance, and patient compliance [130].

Framework exposure deserves specific consideration during maintenance. Although no validated PSSI-specific exposure classification is currently available, minimal asymptomatic stable exposure can be managed conservatively—reinforcement of home care, targeted professional debridement, prosthetic adjustment to improve access, and photographic monitoring [25,32,46]—whereas progressive exposure, persistent inflammation, suppuration, recurrent swelling, pain, ulceration, increasing plaque retention or suspected instability should prompt further assessment. Imaging may be required when deep infection, fixation screw loosening, framework displacement or bone changes are suspected, and treatment escalation may include prosthetic modification, local debridement, soft-tissue management, coverage procedures, or partial or complete implant removal in cases of recurrent infection or mechanical instability [130].

Overall, maintenance should be regarded as an integral part of subperiosteal implant therapy rather than a postoperative accessory. Long-term success depends not only on implant survival and mechanical stability, but also on whether the rehabilitation remains biologically maintainable, accessible to hygiene, compatible with the patient’s functional capacity, and adaptable to changes in soft tissue conditions over time. Future studies should therefore report not only implant survival and complications, but also prosthetic cleanability, recall intervals, home-care instructions, professional hygiene procedures, prosthesis removal protocols, exposure grading, and patient compliance.

## 9. Proposed Clinical Decision Framework

**Figure 3:**
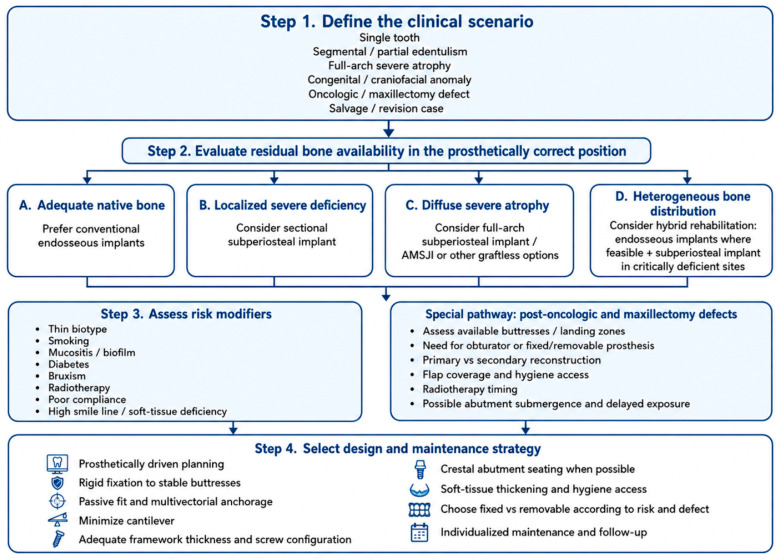
Proposed clinical decision framework for PSSI rehabilitation. The framework represents the authors’ expert interpretation derived from the evidence mapping and is not an evidence-graded recommendation.

Because this scoping review did not include a risk-of-bias assessment or certainty grading, the framework proposed in this section and summarized in Figure 3 should be read as the authors’ expert interpretation derived from the evidence map, rather than as an evidence-graded clinical recommendation. Within these limits, the available evidence suggests that contemporary custom-made subperiosteal implants should not be interpreted as a single therapeutic solution for all forms of jaw atrophy, but rather as a family of patient-specific devices whose indication depends on defect morphology, residual bone distribution, soft tissue quality, prosthetic objectives, and patient-related risk factors [Figure 3]. A clinically useful decision framework should therefore begin with the type of edentulism or defect rather than with the implant itself. Single-tooth agenesis, localized posterior atrophy, full-arch severe edentulism, congenital craniofacial deformities, post-oncologic maxillectomy defects, post-infective or medication-related acquired defects, and salvage cases after failed conventional or zygomatic implants represent distinct clinical scenarios, each requiring different design principles and different success endpoints [14,15,56,59,113].

In patients with sufficient residual native bone in the planned prosthetic position, conventional endosseous implants should remain the reference option, given their extensive long-term evidence base and well-established success criteria. PSSI become more relevant when the residual bone volume is insufficient for predictable endosseous implant placement without major grafting, when the patient refuses or is not suitable for regenerative procedures, or when alternative graftless solutions would result in unfavorable prosthetic emergence, excessive cantilever, sinus-related morbidity, or unacceptable surgical risk [25,33,55]. In this perspective, the indication for a subperiosteal implant should not be based merely on the presence of atrophy, but on whether a patient-specific extraosseous framework can provide a more conservative, prosthetically driven, and maintainable solution than grafting, short implants, pterygoid implants, zygomatic implants, nerve lateralization, or conventional removable prosthodontics.

A site-specific approach appears particularly important in partially edentulous patients. Localized posterior mandibular or maxillary atrophy may be managed with sectional subperiosteal implants when the defect is too severe for short implants or conventional GBR and when the position of zygomatic or pterygoid implants would be incompatible with a limited prosthetic span [25,31,33,70]. Similarly, in single-tooth or anterior esthetic indications, such as lateral incisor agenesis, the rationale is different from full-arch rehabilitation: the goal is not simply to bypass generalized atrophy, but to provide a prosthetically correct emergence profile in a region where congenital bone deficiency, previous GBR failure, or thin soft tissues may make endosseous implants unreliable or esthetically compromised [35]. These indications should be considered highly selective and require careful evaluation of smile line, soft tissue phenotype, adjacent teeth, hygiene access, and long-term esthetic stability.

When residual bone availability is heterogeneous within the same jaw, a hybrid strategy combining endosseous implants in favorable sites and custom-made subperiosteal implants in critically deficient regions may represent the most rational approach. This avoids the unnecessary use of a less established device in areas where conventional implant therapy is feasible, while still allowing graftless rehabilitation of regions where endosseous implants would require extensive augmentation. In such cases, the subperiosteal framework should remain structurally autonomous and rigidly fixed to stable buttresses, rather than depending mechanically on the endosseous implants [32,108]. This principle is relevant not only biomechanically, but also from an evidence-based and medico-legal perspective: the clinician should use the most validated solution where it is anatomically possible and reserve patient-specific subperiosteal components for sites where they provide a clear biological or prosthetic advantage.

Post-oncologic and maxillectomy defects require a separate decision pathway. In these patients, subperiosteal implants are evolving from dental rehabilitation devices into reconstructive platforms that can combine skeletal fixation, soft tissue support, obturator or prosthetic retention, and immediate or delayed oral rehabilitation. In secondary reconstruction, the key question is whether stable residual buttresses, zygomatic remnants, pterygoid regions, skull-base extensions, or reconstructed bone segments can provide sufficient anchorage for a patient-specific framework [38,43,73,101]. In primary reconstruction, digital planning must integrate oncologic margins, osteotomy lines, flap design, radiotherapy timing, prosthetic emergence, and the possibility of abutment submergence or delayed exposure [37,39,40,41]. In irradiated or scarred fields, the indication should be more cautious and must depend on the availability of robust vascularized soft tissue coverage and realistic hygiene access.

Risk stratification should be performed before finalizing the indication. Thin mucosal phenotype, mucositis, poor plaque control, smoking, diabetes, bruxism, previous radiotherapy, xerostomia, complex flap anatomy, high smile line, limited vestibular depth, poor manual dexterity, and low compliance may all increase the probability of recession, exposure, inflammation, prosthetic complications, or failure [46,47,48]. These factors do not necessarily contraindicate treatment, but they should modify the design and maintenance plan. High-risk patients may require wider hygiene spaces, smoother transmucosal surfaces, greater soft tissue thickening, reduced cantilever, removable rather than fixed prosthetic designs, shorter recall intervals, and more conservative counseling regarding expected outcomes.

Once the indication is confirmed, implant design should be prosthetically driven and biologically maintainable. The abutment position should be derived from the diagnostic wax-up, but the framework must respect biomechanical principles: passive fit, rigid fixation, multivectorial anchorage, load distribution over stable buttresses, avoidance of excessive cantilever, sufficient framework thickness, adequate number and diameter of screws, and soft tissue access for long-term hygiene [19,54,58]. In the maxilla, anchorage may involve the nasomaxillary pillar, zygomaticomaxillary buttress, canine pillar, palate, pterygoid region, zygomatic arch root, or skull-base extensions depending on the defect. In the mandible, fixation usually requires careful use of the external oblique ridge, lateral cortex, basal bone, and regions away from the mental nerve and inferior alveolar canal [34,43,85]. The choice of prosthetic design should be integrated at this stage, because a technically successful framework may still fail clinically if the prosthesis prevents cleaning or overloads the transmucosal components.

Finally, patient counseling should explicitly distinguish survival from success. The patient should be informed that contemporary subperiosteal implants show encouraging short- and mid-term survival, but long-term evidence remains limited and heterogeneous. Minor asymptomatic exposure may not necessarily constitute failure, whereas progressive exposure, suppuration, mobility, recurrent infection, pain, or loss of prosthetic function should be considered clinically significant complications [23,28,102]. The final treatment plan should therefore include not only the surgical and prosthetic workflow, but also a predefined maintenance strategy, photographic monitoring, exposure grading, hygiene instruction, and criteria for escalation or revision.

## 10. Discussion

The present review shows that contemporary PSSI should be interpreted neither as a simple revival of historical cast subperiosteal implants nor as a homogeneous alternative to conventional endosseous implantology. Their current role is better understood as a patient-specific, prosthetically driven, fixation-based reconstructive strategy that integrates digital planning, rigid anchorage, prosthetic design, soft-tissue management, and long-term maintenance [14,61,113,128].

The main clinical implication is that evidence depth varies markedly across indications. Full-arch rehabilitation currently represents the most mature application, whereas sectional, oncologic, salvage, congenital, and single-tooth indications remain progressively less supported by robust clinical evidence. This gradient is important because pooling all indications together may overestimate predictability and obscure indication-specific risks. Across indications, survival should be interpreted as a minimum endpoint rather than as proof of durable clinical success, particularly when recurrent maintenance needs, exposure, inflammation, prosthetic compromise, or revision procedures are present.

The dominant biological challenge is the transmucosal and soft-tissue interface. Modern CAD/CAM and additively manufactured frameworks may overcome many limitations of historical devices, but they do not eliminate the risk of mucosal recession, biofilm accumulation, exposure, mucositis, or chronic inflammation. Therefore, long-term success depends not only on passive fit and rigid fixation, but also on soft-tissue phenotype, cleanability, prosthetic access, patient hygiene, and structured maintenance.

Biomechanical, material, and surface-biology data support the same practical principle: PSSI design should remain prosthetically driven, rigidly fixated to stable buttresses, mechanically autonomous, and optimized for both load distribution and hygiene access. Titanium and Ti6Al4V remain the clinical reference standards, whereas polymeric or hybrid materials and surface modifications remain promising but incompletely validated. These preclinical and finite element data should therefore guide design refinement, but should not be overinterpreted as proof of superior long-term clinical performance.

The oncologic and reconstructive literature may represent one of the most transformative areas for future development. In maxillectomy defects, PSSI are no longer only dental supports; they can become multifunctional reconstructive platforms, combining skeletal fixation, obturator or fixed prosthetic retention, soft-tissue support, and immediate or delayed oral rehabilitation [37,38,39,40,41,73]. This is a major conceptual advancement. In selected patients, especially elderly or medically compromised individuals, patient-specific PSSI may offer a route to functional rehabilitation when vascularized bone transfer, zygomatic implants, or conventional endosseous implants are not feasible. Nevertheless, these cases are highly individualized, often involve irradiated or scarred tissues, and require multidisciplinary planning. In this setting, prosthetic retrievability, hygiene access, soft-tissue coverage, and radiotherapy timing may be as important as implant survival.

Several limitations affect the current evidence base. Most clinical studies are retrospective, small, heterogeneous, and limited to short- or mid-term follow-up; many are case reports or technical notes. Comparative and randomized studies remain rare, while longer-term data are limited and less uniformly positive [97,105,107]; consistent with this, a recent meta-analysis reported a decline in pooled survival from 97.8% at ≤3 years to 54.1% in the longest available (6-year) cohort [63]. Outcome definitions, occlusal risk, patient-reported outcomes, and maintenance protocols are inconsistently reported. These gaps prevent firm conclusions about superiority over bone grafting, zygomatic implants, short implants, or conventional implant solutions. Because this was a scoping review, no formal risk-of-bias assessment or certainty grading was performed; therefore, the findings should be interpreted as evidence mapping and hypothesis generation rather than as graded recommendations. In addition, the review protocol, although written before the searches commenced, was not prospectively registered; in line with PRISMA-ScR guidance, this is acknowledged as a limitation, as the absence of a pre-registered protocol may increase the risk of post hoc deviations in the mapping and charting process. Another limitation is the partial non-independence of the primary clinical evidence, because several studies—especially in emerging indications—originate from research groups represented among the authors of this review. These studies were retained because they are clinically relevant in a small and evolving field, but their findings were interpreted cautiously and should be confirmed by independent prospective cohorts. Future research should therefore move from proof-of-concept reporting to indication-specific prospective studies. Separate evidence pathways are needed for full-arch atrophy, segmental posterior defects, single-tooth agenesis, congenital deformities, post-oncologic reconstruction, and salvage cases. Studies should include standardized definitions of survival and success, validated or prospectively defined severity-based reporting of framework exposure, systematic recording of mucositis and bleeding, patient-reported outcomes, prosthetic complications, maintenance protocols, and radiological assessment of bone remodeling. Biomechanical studies should be validated against clinical outcomes and fatigue testing, while material and surface studies should avoid premature clinical claims without histologic or long-term evidence.

## 11. Conclusions

Contemporary custom-made PSSI represent a promising fixation-based, graftless reconstructive option for selected patients with severe anatomical limitations, localized bone deficiency, congenital or craniofacial deformities, salvage needs, or post-ablative defects. Their main value is not to replace conventional implantology, but to expand reconstructive and prosthetic options when endosseous implants, bone grafting, nerve lateralization, sinus augmentation, zygomatic implants, or other established solutions are not feasible, accepted, or prosthetically appropriate.

The evidence is currently most mature for full-arch rehabilitation of severely atrophic jaws and progressively less robust for sectional, oncologic, congenital, salvage, and single-tooth applications. Therefore, indication-specific interpretation is essential. Reported short- and mid-term survival is generally favorable, but survival should not be equated with success. Future outcome reporting should distinguish implant survival, prosthetic survival, biological success, mechanical success, patient-reported benefit, maintenance burden, and true failure. In particular, framework exposure should be reported according to extent, symptoms, inflammation, progression, prosthetic impact, and association with mobility or infection rather than as a simple binary outcome.

From a clinical perspective, PSSI should remain highly selective, prosthetically driven, biologically cautious, and maintenance-oriented. Appliance design should prioritize passive fit, rigid multivectorial fixation to stable buttresses, avoidance of unnecessary cantilevers, retrievability, fixation-screw accessibility, prosthetic cleanability, and soft-tissue compatibility. In hybrid rehabilitations, conventional endosseous implants should be preferred wherever native bone allows predictable placement, while the subperiosteal component should be reserved for critically deficient sectors and designed to remain mechanically autonomous.

Future research should move from feasibility reporting to indication-specific prospective studies and independent multicenter registries. Priority areas include validation of standardized success criteria, prospectively defined exposure-severity reporting, long-term radiological assessment of bone remodeling and bone apposition, fatigue and screw-stability testing, comparison of titanium, PEEK/PEKK, and hybrid materials, evaluation of selective surface treatments for bone-facing and transmucosal regions, and assessment of prosthetic complications and revision burden. Patient follow-up should be standardized, with risk-based recall intervals, professional hygiene protocols adapted to transmucosal frameworks, photographic documentation of soft-tissue changes, monitoring of mucositis and bleeding, prosthetic access evaluation, and systematic patient-reported outcome measures. Only through longer follow-up, independent validation, and standardized reporting will it be possible to determine which PSSI indications are predictable clinical options and which remain experimental or salvage solutions.

## Figures and Tables

**Table 1:** Evidence on full-arch rehabilitation of severely atrophic edentulous jaws.

Author, Year	Study Type	Indication/Population	Jaw/Site	No. Patients/Implants	Follow-Up	Main Outcomes	Reporting Level
Łoginoff et al., 2026 [105]	Long-term retrospective study	DMLS PSSI in severe mandibular atrophy Cawood–Howell IV–VI	Mandible	10 patients	Up to 12 years	Survival 70%; 3/10 removed due to late recurrent infections, mandibular resorption, and granulation tissue; no soft-tissue dehiscence	Patient + implant
Milad et al., 2026 [98]	Case report	Elderly patient with Cawood VI maxillary atrophy treated with custom 3D-printed PSSI	Maxilla	1 patient	1 year	100% survival; VAS satisfaction 9.5/10; no complications	Patient
Łoginoff et al., 2025 [97]	Long-term retrospective study	DMLS custom PSSI in severely atrophic maxilla	Maxilla	10 patients	Up to 10 years	8/10 implants functional; 2 removed due to poor fit and recurrent infections; Kaplan–Meier survival 80% at 10 years	Implant
Van den Borre et al., 2025 [106]	Multicentre retrospective study	Severe mandibular atrophy, Cawood–Howell IV–VI, treated with PSSI	Mandible	19 patients/40 implants	Mean 804 days	Survival 92.5%; high patient satisfaction; mean OHIP-14 6.68 and NRS 51.26; immediate oedema and temporary infection were the most common adverse events; 2 implants removed for persistent infection/suppuration and 1 scheduled for removal; 1 implant showed mobility > 1 mm; mucosal recession in 13 implants, 32.5%, not perceived by patients as functional or aesthetic concern	Implant
Vaira et al., 2024 [25]	Multicenter retrospective study	Full-arch rehabilitation of severely atrophic maxilla with additively manufactured custom PSSI	Maxilla	36 patients/72 implants	Up to 4 years	No implant loss; success rate 90.3%; limited asymptomatic framework exposure in 9.7%; no significant bone resorption beneath abutments	Implant
Gasparini et al., 2024 [24]	Retrospective clinical study	CAD-CAM subperiosteal implants for atrophic jaws	Maxilla and mandible	18 patients	1 year	Short-term outcomes after digital CAD-CAM rehabilitation	Patient
El-Sawy et al., 2024 [52]	Clinical case series	Full-arch maxillary rehabilitation using PEEK subperiosteal framework	Maxilla	4 patients	1 year	Functional stability; healthy tissues; no infection, pus, mobility, exposure, or prosthetic fracture	Patient
Onică et al., 2024 [107]	Long-term clinical study	PSSI in severe jaw atrophy	Maxilla and mandible	36 patients/61 implants	6 years	Only 9/36 cases considered successful; 27 cases had complications, including exposure, mobility, infection, and progressive framework exposure	Patient (success) + implant
Van den Borre et al., 2024 [46]	Multicenter clinical study	Soft tissue response after bilateral PSSI in severe maxillary atrophy	Maxilla	40 patients	Mean 917 days	Framework exposure/recession in 65%; thin biotype and mucositis significant risk factors; smoking OR 6.88, not statistically significant	Patient
Dimitroulis et al., 2022 [23]	Cohort case series	New-generation CAD-CAM/3D-printed PSSI for atrophic edentulous jaws	Maxilla and mandible	21 implants	Short- to mid-term	Primary success 66.7%; complications in 7 cases, mainly framework exposure; 4 cases salvaged, increasing overall success to 85.7%	Implant
Nemtoi et al., 2022 [18]	Prospective pilot study	Severe bone-deficient patients treated with DMLS titanium custom PSSI	Maxilla and mandible	16 patients/16 implants	Short-term	Mean fit score 4/5; mean operative time 86.18 min; one implant lost due to insufficient fit and recurrent infections	Implant
Van den Borre et al., 2022 [22]	Prospective multicenter study	Severe maxillary atrophy Cawood–Howell ≥ V treated with PSSI	Maxilla	15 patients	1 year	OHIP-14 improved from 17.20 to 5.80; satisfaction exceeded expectations; no complications reported in short-term follow-up	Patient
Van den Borre et al., 2021 [95]	Radiographic follow-up study	Severe maxillary atrophy treated with PSSI	Maxilla	15 patients	1 year	Mean negative crestal remodeling 0.26 mm; minimal bone loss around supporting bone, wings, and basal frame; no significant radiographic crestal atrophy	Implant
Cerea and Dolcini, 2018 [17]	Retrospective case series	Elderly patients with severe jaw atrophy rehabilitated with DMLS titanium PSSI	Maxilla and/or mandible	70 patients	Minimum 2 years	Survival 95.8%; 3 implants lost due to recurrent infections; immediate complications 5.7%, biological complications 1.4%, prosthetic complications 8.9%	Implant
Mounir et al., 2018 [69]	Prospective clinical study	Severe anterior maxillary atrophy treated with patient-specific titanium or PEEK subperiosteal implants	Maxilla	10 patients/10 implants	12 months	All implants stable; one dehiscence in titanium group; no resorption, mobility, infection, or prosthetic fracture	Implant

**Table 2:** Evidence on segmental/sectional rehabilitation of partially edentulous jaws.

Author, Year	Study Type	Indication/Population	Jaw/Site	No. Patients/Implants	Follow-Up	Main Outcomes	Reporting Level
Vaira et al., 2026 [32]	Retrospective case series	Hybrid rehabilitation combining PSSI and conventional endosseous implants in heterogeneous bone availability	Maxilla and mandible	14 patients/20 PSSI + 48 endosseous implants	Mean 22.1 months	100% survival for both implant systems; 100% rehabilitation survival; minor manageable complications	Patient + implant
Vaira et al., 2025 [33]	Cohort study	Cawood–Howell V–VI posterior maxillary atrophy in partially dentate patients	Posterior maxilla	16 patients/21 implants	Median 36 months	Survival and success 95.2% at 1 and 5 years; mean bone resorption beneath abutments 0.18 mm at 1 year; BOP decreased from 10% at 6 months to 2.5% at 4 years; no framework exposure	Implant
Pellegrino et al., 2025 [21]	Case series	PSSI for several indications including narrow crests and partial atrophy	Maxilla and mandible	9 patients/11 sites	Mean 36.2 months	Minor complications in 3 patients; no early failure, screw loosening, prosthetic fracture, or framework exposure	Patient
Darwish et al., 2025 [104]	Randomized clinical trial	Milled vs. 3D-printed PSSI in severe mandibular atrophy	Mandible	20 patients	1 year	Survival 100% in 3D-printing group vs. 90% in milling group; no significant differences in survival, bone resorption, accuracy, operative time, or dehiscence	Patient + implant
Cariati et al., 2025 [96]	Retrospective case series	Virtual-planned maxillary PSSI in severe Cawood–Howell V–VI atrophy	Maxilla	12 patients	3 years	Survival 91.7%; complications in 4/12, including 2 exposures, bleeding in anticoagulated patient, and 1 failure related to insufficient bone reshaping	Patient
Vaira et al., 2024 [34]	Cohort study	Severe posterior mandibular atrophy treated with additively manufactured custom PSSI	Posterior mandible	17 patients/30 implants	Mean 22.5 months	No exposure, infection, implant loss, screw loosening, or displacement; moderate edema was main postoperative sequela	Implant
Vaira et al., 2024 [36]	Case report	Severe maxillary molar atrophy where sinus lift was refused and zygomatic/pterygoid implants were unsuitable	Posterior maxilla, molar sector	1 patient/1 implant	38 months	Temporary prosthesis at 10 days; definitive zirconia at 6 months; no clinical or radiological complications	Implant
Gellrich et al., 2024 [108]	Case series	Complex bimaxillary cases treated with PSSI combined with conventional implants	Bimaxillary/complex defects	4 patients/5 PSSI + 20 conventional implants	Up to 68 months	No failures or loosening; multivectorial fixation with 13–22 screws	Implant
Nedelcu et al., 2024 [70]	Case report	Severe posterior mandibular atrophy after previous implant complication; residual bone 4.5–5 mm above IAN	Posterior mandible	1 patient/1 implant	Short-tem	Custom 3D-printed PSSI proposed as alternative to short implants, grafting or IAN lateralization	Implant
Fathi et al., 2024 [109]	Case report	Severe mandibular atrophy rehabilitated with PSSI supporting overdenture	Mandible	1 patient/1 implant	1 year	High satisfaction; no complications; fixation with 19 screws	Implant
Arshad et al., 2024 [110]	Case report	Existing mandibular PSSI used for overdenture support after mandibular fracture management	Mandible	1 patient	1 year	Good outcome; prosthetic rescue with connector to manage submerged ball abutments	Patient
Krishnaprabhu et al., 2021 [111]	Prospective clinical evaluation	Hybrid implants in posterior maxillary rehabilitation	Posterior maxilla	27 patients/30 implants	1 year	100% survival; 6.7% exposure; 13.3% instability; physiological bone loss reported	Implant
Mangano et al., 2020 [31]	Case series	Elderly patients with atrophic posterior mandible	Posterior mandible	10 patients/10 implants	1 year	Survival 100%; minor complications in 30%, including pain/swelling and provisional prosthesis fractures	Implant

**Table 3:** Evidence on congenital, syndromic, and craniofacial indications.

Author, Year	Study Type	Indication/Population	Jaw/Site	No. Patients/Implants	Follow-Up	Main Outcomes	Reporting Level
De Riu et al., 2025 [71]	Case report	EEC syndrome with cleft-related deformity, maxillary hypoplasia, severe residual atrophy, and previous reconstructive procedures	Maxilla	1 patient/2 PSSI	18 months	Stable implants; no exposure; no BOP; no soft-tissue or prosthetic complications	Implant
Wirth et al., 2025 [72]	Case report	Unrepaired cleft palate, severe maxillary deformity, chronic sinusitis, previous failure of conventional and zygomatic implants	Maxilla	1 patient/2 PSSI combined with residual tuberosity implants	Short-term follow-up at time of report	No complications reported; functional obturator support after multiple previous failures	Implant
Debortoli et al., 2025 [99]	Case report	Young patient with skeletal class III, maxillary deformity, endognathia, and compromised dentition requiring combined skeletal and prosthetic correction	Maxilla	1 patient/PSSI dual-block with 6 abutments	6 months	No dehiscence or infection; correction from class III to class I; improved esthetic and occlusal relationship	Implant/abutment
Ângelo and Ferreira, 2020 [77]	Case report	Dental agenesis with previous failure of mandibular all-on-6 rehabilitation due to peri-implantitis	Bimaxillary rehabilitation	1 patient/1 PSSI	Short-term follow-up reported	Proposed as primary or rescue option after failed conventional implantology	Implant

**Table 4:** Evidence on post-oncologic, post-ablative, and maxillectomy reconstruction.

Author, Year	Study Type	Indication/Population	Jaw/Site	No. Patients/Implants	Follow-Up	Main Outcomes	Reporting Level
Frias et al., 2026 [42]	Case report	Previous SCC with oroantral fistula and compromised residual dentition	Maxilla/oroantral defect	1 patient/1 PSSI	6 months	Custom maxillary PSSI retained immediate surgical obturator; later implant-retained removable obturator; mild exposure near fistula	Implant
De Riu et al., 2025 [39]	Case report	SCC of hard palate treated with total maxillectomy, fibula free flap, and custom PSSI	Total maxillectomy + fibula free flap	1 patient /1 PSSI	2 years	Digital planning, production in ~10 days; abutments submerged during RT and uncovered later; definitive prosthesis at 6 months; no complications; disease-free	Implant
De Riu et al., 2025 [37]	Retrospective case series	Primary maxillary reconstruction during oncologic surgery using 3D-printed custom PSSI	Maxillary oncologic defects	9 patients/9 implants	6–20 months; mean 13.7 months	All implants placed and loaded; no infection or mobility; no prosthetic complications; one flap necrosis unrelated to implant; one score 1A exposure; severe mucositis in one irradiated patient	Implant
Gellrich et al., 2025 [43]	Case series	Complex maxillary/midfacial defects requiring innovative landing zones	Maxilla/midface; skull base/pterygoid extensions	13 patients/13 implants	9–52 months; mean 37.5 months	Immediate stability and prosthetic restoration in all; no stability loss/peri-implantitis; one removal for pain/infection in text	Implant
Segna et al., 2025 [40]	Case report	One-step reconstruction after maxillectomy for benign tumor	Maxilla/hemimaxillectomy	1 patient/1 PSSI	12 months	Immediate prosthetic rehabilitation; satisfactory facial symmetry, stable occlusion, bone healing, and no infection or hardware failure.	Implant
John et al., 2025 [112]	Case report	MRONJ-related jaw defect after nintedanib requiring resection and rehabilitation	Acquired jaw defect	1 patient/1 PSSI	6 months	Patient-specific titanium implant; fixed prosthesis; 1–2 mm superficial exposure without instability or prosthetic compromise	Implant
Basavaraju et al., 2024 [75]	Case report	Subtotal unilateral maxillectomy after post-COVID mucormycosis	Maxilla/zygomatic support	1 patient/1 PSSI	Short-term	DMLS titanium framework on zygomatic contour with fixed prosthetic rehabilitation	Implant
Surana et al., 2024 [76]	Case report	Bilateral low-level maxillectomy after post-COVID mucormycosis	Maxilla/zygomatic support	1 patient/1 PSSI	6 months	Right PSSI infected and removed; subsequent overdenture on CAD/CAM Hader bar	Implant
De Riu et al., 2023 [41]	Case report	Elderly, comorbid patient with palatal/maxillary SCC not suitable for free flap	Total maxillectomy	1 patient/1 PSSI	6 months	Implant produced in 9 days; fixation to nasomaxillary pillars and zygomas; supported soft tissues and prosthesis/obturator; dehiscence/fistula related to temporalis fascia necrosis, managed with obturator	Implant
Cebrián Carretero et al., 2022 [38]	Case series	Maxillary defects after oncologic resection treated with customized subperiosteal titanium maxillary implants	Maxilla	4 patients/4 implants	Short-term	VSP, STL models, CAD/CAM titanium mesh, fixed prosthetic rehabilitation; good esthetic-functional outcomes	Implant
Kondaka et al., 2022 [74]	Case report	Post-COVID mucormycosis with bimaxillary/maxillary resection defect	Maxilla/zygomatic support	1 patient/1 PSSI	Short-term	PSSI supported by zygomatic remnants improved speech, swallowing, and function	Implant
Garrido-Martínez et al., 2022 [100]	Case report	Maxillary SCC treated with resection and subsequent custom subperiosteal implant rehabilitation	Maxilla	1 patient/1 PSSI	1 year	Sintered titanium implant and customized prosthetic rehabilitation after oncologic surgery	Implant
Vosselman et al., 2019 [73]	Case report	Post-oncologic maxillary defect after subtotal bilateral maxillectomy for palatal SCC	Zygoma-supported maxillary defect	1 patient/1 PSSI	Short-term follow-up at time of report	Patient-specific subperiosteal zygoma implant used to support obturator; improved speech and swallowing without nasal leakage	Implant

**Table 5:** Evidence on rescue and salvage indications.

Author, Year	Study Type	Indication/Population	Jaw/Site	No. Patients/Implants	Follow-Up	Main Outcomes	Reporting Level
Revuelta-Cortés et al., 2026 [79]	Case report	Severe maxillary atrophy after implant failure/peri-implantitis	Maxilla	1 patient	Short-term	Digital CBCT + CAD/CAM workflow; PSSI as alternative to bone grafting	Patient + implant
Cardoso and Grillo, 2025 [78]	Case report	Failed/sequelae of bilateral zygomatic implants	Maxilla	1 patient	>1 year	Removal of zygomatic implants and placement of a PSSI; improved quality of life	Patient + implant
Wirth et al., 2025 [72]	Case report	Unrepaired cleft palate with multiple failed endosseous and zygomatic implants, chronic sinusitis, bone loss, and palatal defect	Maxilla	1 patient/2 PSSI + residual tuberosity implants	Short-term	Implant-retained obturator; no complications reported	Implant
Parras-Hernández et al., 2024 [114]	Case report	Severe maxillary defect secondary to rhino-orbit-cerebral mucormycosis after failed fibula free flap reconstruction	Maxilla	1 patient/1 personalized PSSI-supported obturator	Short-term	Personalized subperiosteal implant-supported obturator used as a salvage solution after failure of conventional reconstructive surgery; functional rehabilitation in a highly complex post-infective defect	Implant
Nedelcu et al., 2024 [70]	Case report	Severe posterior mandibular atrophy after prior implant failure/complication; limited residual bone above IAN	Posterior mandible	1 patient	Short-term	PSSI proposed instead of short implants, grafting, or IAN lateralization	Patient + implant
Ângelo and Ferreira, 2020 [77]	Case report	Dental agenesis and failed mandibular all-on-6 rehabilitation due to peri-implantitis	Bimaxillary	1 patient	Short-term	Custom bimaxillary subperiosteal implants with combined subperiosteal/endosseous concept	Patient + implant

**Table 6:** Finite element and biomechanical studies on custom-made subperiosteal implants.

Author, Year	Anatomical Model/Indication	Design or Comparison Evaluated	Main Biomechanical Findings
Vörös et al., 2025 [116]	In vitro mechanical screw-loosening model	Tightening torque of M1.8 connection screws: 15 Ncm vs. 30 Ncm after cyclic loading	15 Ncm did not provide sufficient stability and led to loosening after cyclic loading; 30 Ncm maintained better stability.
Demir and Caglar, 2025 [51]	Full-arch maxillary subperiosteal implant	Prosthetic framework materials: CoCr-porcelain, Ti-porcelain, Ti-acrylic, Zr-porcelain, PEEK-composite	PEEK-composite produced higher stress in bone, implant, and screws; CoCr and zirconia showed more favorable biomechanical behavior; prosthetic screws were the highest-stress area.
Canko and Doganay Ozyilmaz, 2025 [50]	PSSI maxillary model	Framework material, cantilever presence, and I-shaped vs. Y-shaped wing design	CoCr, I-shaped configuration, and absence of cantilever showed more favorable stress distribution; cantilevers increased displacement and stress; PEEK reduced framework stress but increased deformation/displacement.
Roshdy et al., 2025 [86]	Atrophic edentulous mandible	Single-piece vs. two-piece titanium subperiosteal implants	Bone, screw, and prosthetic stresses were similar, but the two-piece framework showed more than double the framework stress compared with the single-piece design.
Vanaclocha et al., 2025 [54]	Maxillary and mandibular subperiosteal implant designs	Iterative FEA optimization plus static and fatigue mechanical testing	Optimized Ti6Al4V laser-powder bed fusion implants resisted static loading at 450 N and fatigue loading at 150 N for 5 million cycles without failure.
Pellegrino et al., 2025 [21]	Realistic models of atrophic jaws	Subperiosteal/juxta-osseous implant models with different screw and load-distribution configurations	Screws, connections, and load distribution strongly influenced stress; additional posterior fixation and broader load distribution reduced critical concentrations.
Deniz & Yurttutan, 2025 [53]	Totally edentulous atrophic maxilla	Conventional + zygomatic implants vs. maxilla-anchored and zygomatic bone-anchored subperiosteal designs	Zygomatic bone-anchored subperiosteal design reduced stress in cortical and trabecular bone, although implant, abutment, and screw stresses increased.
El-Sawy et al., 2025 [80]	Atrophic maxilla	Titanium, modified PEEK/BioHPP, and PEKK combinations for framework and superstructure	Titanium framework transferred less stress to bone and screws and showed better stability under full-arch loading; PEEK/PEKK could increase stress in cement layer, framework, or bone under anterior loading.
Arı & Acar, 2025 [117]	Total maxillectomy defect, Liverpool Class II	Conventional subperiosteal design vs. alternative design with diagonal zygomatic bar	The diagonal bar design did not provide a clear biomechanical advantage and showed higher stresses under oblique loading in some regions.
Baş et al., 2025 [118]	Severely atrophic edentulous mandible under traumatic anterior force	IAN lateralization + 6 implants, All-on-four, PEEK subperiosteal implant, titanium subperiosteal implant	All-on-four and PEEK subperiosteal implant behaved more favorably under traumatic loading than titanium PSSI and IAN lateralization models; PEEK showed more balanced stress on abutments and screws.
Parhiz et al., 2024 [85]	Severely atrophic mandible	Novel modular mandibular patient-specific implant with inferior border cover and horseshoe component	Stress concentrated mainly around screw sites; increasing the number and distribution of fixation screws reduced loosening risk.
Acar et al., 2024 [84]	Unilateral maxillary defect	Subperiosteal implants vs. zygomatic implant-based configurations	Subperiosteal designs transmitted less stress to alveolar bone than zygomatic configurations; one-piece PSSI showed favorable distribution.
Kundakcioglu and Gedik, 2024 [119]	Custom subperiosteal implant model	Fixation screws of 1.5 mm vs. 2.0 mm diameter	Screws of 2.0 mm reduced stress on bone and implant components, while 1.5 mm screws showed different movement patterns.
Kundakcioglu and Ayhan, 2024 [49]	Subperiosteal implant model under 250 N load	Framework thickness of 1.0, 1.5, and 2.0 mm	The 1.0 mm design exceeded yield limits and showed plastic deformation; 1.5 mm appeared a more reasonable minimum thickness in the tested model.
Arı et al., 2024 [83]	Total maxillectomy defect	Quad zygoma, zygoma + partial subperiosteal implants, one-piece and two-piece PSSI combinations	Combined zygomatic–subperiosteal strategies, especially selected two-piece PSSI scenarios, produced more balanced stress distribution in several components.
Zielinski et al., 2023 [81]	Edentulous maxilla; MaI Implant	Single implant vs. two implants connected by a bar; different load magnitudes and directions	Displacement increased with higher loads and with more oblique loading angles; stabilization and load direction strongly influenced mechanical behavior.
Castrillo et al., 2023 [82]	Subperiosteal implant screw fixation model	Advanced FEA submodelling of screw-to-bone interaction	Modelling the threaded screw–bone interface changed displacement and stress patterns; cortical bone was the most relevant region for stress concentration.
Ayhan and Cankaya, 2023 [26]	Atrophic maxilla	Monoblock vs. dual custom subperiosteal implant systems	Dual geometry produced lower stress values than monoblock designs; displacement remained low under static loading.
Cipollina et al., 2023 [120]	Severely atrophic maxilla/premaxillary region	Premaxillary device as a subperiosteal or hybrid implant concept	FEA suggested favorable stress distribution on basal bone and values within resistance limits of bone and titanium alloys.
De Moor et al., 2022 [19]	Severely atrophic maxilla; patient-specific AMSJI	Mechanical evaluation of a patient-specific AMSJI under functional loading	The implant appeared mechanically safe under average occlusal forces, but higher stresses were concentrated in the arms and may become critical in extreme atrophy, bruxism, or maximal occlusal loading.
Altıparmak et al., 2022 [121]	Atrophic maxilla	Titanium vs. 60% carbon-fiber-reinforced PEEK	Titanium showed higher stress within the implant system, whereas stresses transmitted to cortical and cancellous bone were broadly similar between titanium and CFR-PEEK.

## Data Availability

No new original data were generated in this study. All data analyzed in this review were derived from previously published articles, which are cited in the reference list. Therefore, data sharing is not applicable to this article.

## Supplementary Materials

The following supporting information can be downloaded at: https://www.mdpi.com/article/10.3390/jcm15135220/s1, Table S1: PRISMA-ScR Checklist; Table S2: Complete electronic search strategies; Table S3. Record-to-category mapping of the 56 unique human clinical records with extractable denominators.
