# Supplementary material for: Patient-Specific Subperiosteal Implants for Oral and Maxillofacial Rehabilitation: A Scoping Review Across Indications, from Established Full-Arch Use to Emerging Single-Tooth and Oncologic Applications
Source: J Clin Med. 2026 Jul 3;15(13):5220. doi: 10.3390/jcm15135220 (PMC13363636; doi:10.3390/jcm15135220)
Supplement: Supplementary file 1 [file jcm-15-05220-s001.zip › jcm-4389258-supplementary.pdf]

Supplementary Table S1. PRISMA-ScR Checklist

Patient-Specific Subperiosteal Implants for Oral and Maxillofacial Rehabilitation: A Scoping Review Across Indications, from Established Full-Arch Use to Emerging Single-Tooth and Oncologic Applications

SECTION	ITEM	PRISMA-ScR CHECKLIST ITEM	REPORTED ON PAGE/SECTION
**TITLE**			
Title	1	Identify the report as a scoping review.	Title page
**ABSTRACT**			
Structured summary	2	Provide a structured summary that includes (as applicable): background, objectives, eligibility criteria, sources of evidence, charting methods, results, and conclusions that relate to the review questions and objectives.	Abstract
**INTRODUCTION**			
Rationale	3	Describe the rationale for the review in the context of what is already known. Explain why the review questions/objectives lend themselves to a scoping review approach.	Introduction, Section 1
Objectives	4	Provide an explicit statement of the questions and objectives being addressed with reference to their key elements (e.g., population or participants, concepts, and context) or other relevant key elements used to conceptualize the review questions and/or objectives.	Introduction, final paragraph; Methods, Section 2.1 (Population-Concept-Context framework)
**METHODS**			
Protocol and registration	5	Indicate whether a review protocol exists; state if and where it can be accessed (e.g., a Web address); and if available, provide registration information, including the registration number.	Methods, Section 2.1 (protocol written but not prospectively registered)
Eligibility criteria	6	Specify characteristics of the sources of evidence used as eligibility criteria (e.g., years considered, language, and publication status), and provide a rationale.	Methods, Section 2.2
Information sources*	7	Describe all information sources in the search (e.g., databases with dates of coverage and contact with authors to identify additional sources), as well as the date the most recent search was executed.	Methods, Section 2.3
Search	8	Present the full electronic search strategy for at least 1 database, including any limits used, such that it could be repeated.	Methods, Section 2.3;
Selection of sources of evidence†	9	State the process for selecting sources of evidence (i.e., screening and eligibility) included in the scoping review.	Methods, Section 2.4
Data charting process‡	10	Describe the methods of charting data from the included sources of evidence (e.g., calibrated forms or forms that have been tested by the team before their use, and whether data charting was done independently or in duplicate) and any processes for obtaining and confirming data from investigators.	Methods, Section 2.5
Data items	11	List and define all variables for which data were sought and any assumptions and simplifications made.	Methods, Section 2.5
Critical appraisal of individual sources of evidence§	12	If done, provide a rationale for conducting a critical appraisal of included sources of evidence; describe the methods used and how this information was used in any data synthesis (if appropriate).	Not applicable; rationale in Methods, Section 2.6
Synthesis of results	13	Describe the methods of handling and summarizing the data that were charted.	Methods, Section 2.6
**RESULTS**			
Selection of sources of evidence	14	Give numbers of sources of evidence screened, assessed for eligibility, and included in the review, with reasons for exclusions at each stage, ideally using a flow diagram.	Results, Section 3.1; Figure 1
Characteristics of sources of evidence	15	For each source of evidence, present characteristics for which data were charted and provide the citations.	Results, Sections 3.2-3.3; Tables 1-6
Critical appraisal within sources of evidence	16	If done, present data on critical appraisal of included sources of evidence (see item 12).	Not applicable; no formal critical appraisal performed
Results of individual sources of evidence	17	For each included source of evidence, present the relevant data that were charted that relate to the review questions and objectives.	Results, Sections 4-8; Tables 1-6
Synthesis of results	18	Summarize and/or present the charting results as they relate to the review questions and objectives.	Results, Sections 3-8
**DISCUSSION**			
Summary of evidence	19	Summarize the main results (including an overview of concepts, themes, and types of evidence available), link to the review questions and objectives, and consider the relevance to key groups.	Discussion, Section 10
Limitations	20	Discuss the limitations of the scoping review process.	Discussion, Section 10, limitations paragraph
Conclusions	21	Provide a general interpretation of the results with respect to the review questions and objectives, as well as potential implications and/or next steps.	Conclusions, Section 11
**FUNDING**			
Funding	22	Describe sources of funding for the included sources of evidence, as well as sources of funding for the scoping review. Describe the role of the funders of the scoping review.	Funding statement (no external funding)

Supplementary Table S2. Complete electronic search strategies

Search date: 1 May 2026. No date restriction was applied during the initial search. Searches were adapted to each database syntax. Records were later categorized as historical or contemporary according to implant concept and manufacturing workflow during study selection.

Database / source	Platform and field tags	Complete search strategy	Limits / filters	Records retrieved
MEDLINE/PubMed	PubMed; [tiab], [MeSH Terms]	(("subperiosteal implant"[tiab] OR "subperiosteal implants"[tiab] OR "subperiosteal jaw implant"[tiab] OR "subperiosteal jaw implants"[tiab] OR AMSJI[tiab] OR "patient-specific subperiosteal implant"[tiab] OR "patient specific subperiosteal implant"[tiab] OR "custom-made subperiosteal implant"[tiab] OR "customized subperiosteal implant"[tiab] OR "CAD/CAM subperiosteal implant"[tiab] OR "3D printed subperiosteal implant"[tiab] OR "additively manufactured subperiosteal implant"[tiab]) OR (("subperiosteal"[tiab]) AND (implant*[tiab] OR framework*[tiab] OR device*[tiab]))) AND (("patient-specific"[tiab] OR "patient specific"[tiab] OR custom*[tiab] OR customized[tiab] OR "custom-made"[tiab] OR CADCAM[tiab] OR "CAD-CAM"[tiab] OR "CAD/CAM"[tiab] OR "computer-aided design"[tiab] OR "computer aided design"[tiab] OR "additive manufacturing"[tiab] OR "3D print*"[tiab] OR "three-dimensional print*"[tiab] OR "direct metal laser sintering"[tiab] OR DMLS[tiab] OR "selective laser melting"[tiab] OR SLM[tiab] OR "laser melting"[tiab] OR milling[tiab]) OR ("Computer-Aided Design"[MeSH Terms] OR "Printing, Three-Dimensional"[MeSH Terms])) AND (("atrophic maxilla"[tiab] OR "atrophic mandible"[tiab] OR "atrophic jaw"[tiab] OR "severe jaw atrophy"[tiab] OR "jaw atrophy"[tiab] OR "bone deficiency"[tiab] OR edentulous[tiab] OR "partial edentulism"[tiab] OR "fixed partial restoration"[tiab] OR "oral rehabilitation"[tiab] OR "dental rehabilitation"[tiab] OR maxillectomy[tiab] OR "maxillary defect"[tiab] OR "mandibular defect"[tiab] OR "midface defect"[tiab] OR "maxillofacial reconstruction"[tiab] OR obturator[tiab] OR cleft[tiab] OR agenesis[tiab]) OR ("Mouth Rehabilitation"[MeSH Terms] OR "Jaw, Edentulous"[MeSH Terms] OR "Maxillofacial Prosthesis"[MeSH Terms]))	Database inception to 1 May 2026; no language, article-type, or publication-date filter applied in the electronic search.	476
Scopus	Scopus; TITLE-ABS-KEY	TITLE-ABS-KEY(("subperiosteal implant" OR "subperiosteal implants" OR "subperiosteal jaw implant" OR "subperiosteal jaw implants" OR AMSJI OR "patient-specific subperiosteal implant" OR "patient specific subperiosteal implant" OR "custom-made subperiosteal implant" OR "customized subperiosteal implant" OR "CAD/CAM subperiosteal implant" OR "3D printed subperiosteal implant" OR "additively manufactured subperiosteal implant" OR (subperiosteal W/3 (implant* OR framework* OR device*))) AND ("patient-specific" OR "patient specific" OR custom* OR customized OR "custom-made" OR CADCAM OR "CAD-CAM" OR "CAD/CAM" OR "computer-aided design" OR "computer aided design" OR "additive manufacturing" OR "3D print*" OR "three-dimensional print*" OR "direct metal laser sintering" OR DMLS OR "selective laser melting" OR SLM OR "laser melting" OR milling) AND ("atrophic maxilla" OR "atrophic mandible" OR "atrophic jaw" OR "severe jaw atrophy" OR "jaw atrophy" OR "bone deficiency" OR edentulous OR "partial edentulism" OR "fixed partial restoration" OR "oral rehabilitation" OR "dental rehabilitation" OR maxillectomy OR "maxillary defect" OR "mandibular defect" OR "midface defect" OR "maxillofacial reconstruction" OR obturator OR cleft OR agenesis))	Database inception to 1 May 2026; no document-type, language, or publication-year filter applied.	196
Web of Science Core Collection	Web of Science; TS=	TS=(("subperiosteal implant" OR "subperiosteal implants" OR "subperiosteal jaw implant" OR "subperiosteal jaw implants" OR AMSJI OR "patient-specific subperiosteal implant" OR "patient specific subperiosteal implant" OR "custom-made subperiosteal implant" OR "customized subperiosteal implant" OR "CAD/CAM subperiosteal implant" OR "3D printed subperiosteal implant" OR "additively manufactured subperiosteal implant" OR (subperiosteal NEAR/3 (implant* OR framework* OR device*))) AND ("patient-specific" OR "patient specific" OR custom* OR customized OR "custom-made" OR CADCAM OR "CAD-CAM" OR "CAD/CAM" OR "computer-aided design" OR "computer aided design" OR "additive manufacturing" OR "3D print*" OR "three-dimensional print*" OR "direct metal laser sintering" OR DMLS OR "selective laser melting" OR SLM OR "laser melting" OR milling) AND ("atrophic maxilla" OR "atrophic mandible" OR "atrophic jaw" OR "severe jaw atrophy" OR "jaw atrophy" OR "bone deficiency" OR edentulous OR "partial edentulism" OR "fixed partial restoration" OR "oral rehabilitation" OR "dental rehabilitation" OR maxillectomy OR "maxillary defect" OR "mandibular defect" OR "midface defect" OR "maxillofacial reconstruction" OR obturator OR cleft OR agenesis))	Database inception to 1 May 2026; no document-type, language, or publication-year filter applied.	143
Embase	Embase; title/abstract fields and Emtree terms	('subperiosteal implant':ti,ab OR 'subperiosteal implants':ti,ab OR 'subperiosteal jaw implant':ti,ab OR 'subperiosteal jaw implants':ti,ab OR amsji:ti,ab OR 'patient-specific subperiosteal implant':ti,ab OR 'patient specific subperiosteal implant':ti,ab OR 'custom-made subperiosteal implant':ti,ab OR 'customized subperiosteal implant':ti,ab OR 'cad/cam subperiosteal implant':ti,ab OR '3d printed subperiosteal implant':ti,ab OR 'additively manufactured subperiosteal implant':ti,ab OR (subperiosteal:ti,ab NEAR/3 (implant*:ti,ab OR framework*:ti,ab OR device*:ti,ab))) AND ('patient-specific':ti,ab OR 'patient specific':ti,ab OR custom*:ti,ab OR customized:ti,ab OR 'custom-made':ti,ab OR cadcam:ti,ab OR 'cad-cam':ti,ab OR 'cad/cam':ti,ab OR 'computer-aided design':ti,ab OR 'computer aided design':ti,ab OR 'additive manufacturing':ti,ab OR '3d print*':ti,ab OR 'three-dimensional print*':ti,ab OR 'direct metal laser sintering':ti,ab OR dmls:ti,ab OR 'selective laser melting':ti,ab OR slm:ti,ab OR 'laser melting':ti,ab OR milling:ti,ab OR 'computer aided design'/exp OR 'three dimensional printing'/exp) AND ('atrophic maxilla':ti,ab OR 'atrophic mandible':ti,ab OR 'atrophic jaw':ti,ab OR 'severe jaw atrophy':ti,ab OR 'jaw atrophy':ti,ab OR 'bone deficiency':ti,ab OR edentulous:ti,ab OR 'partial edentulism':ti,ab OR 'fixed partial restoration':ti,ab OR 'oral rehabilitation':ti,ab OR 'dental rehabilitation':ti,ab OR maxillectomy:ti,ab OR 'maxillary defect':ti,ab OR 'mandibular defect':ti,ab OR 'midface defect':ti,ab OR 'maxillofacial reconstruction':ti,ab OR obturator:ti,ab OR cleft:ti,ab OR agenesis:ti,ab OR 'jaw reconstruction'/exp OR 'mouth rehabilitation'/exp)	Database inception to 1 May 2026; no document-type, language, or publication-year filter applied.	77

Cochrane Library	Cochrane Library; Title Abstract Keyword	(("subperiosteal implant" OR "subperiosteal implants" OR "subperiosteal jaw implant" OR "subperiosteal jaw implants" OR AMSJI OR "patient-specific subperiosteal implant" OR "patient specific subperiosteal implant" OR "custom-made subperiosteal implant" OR "customized subperiosteal implant" OR "CAD/CAM subperiosteal implant" OR "3D printed subperiosteal implant" OR "additively manufactured subperiosteal implant") OR (subperiosteal NEAR/3 (implant* OR framework* OR device*))) AND ("patient-specific" OR "patient specific" OR custom* OR customized OR "custom-made" OR CADCAM OR "CAD-CAM" OR "CAD/CAM" OR "computer-aided design" OR "computer aided design" OR "additive manufacturing" OR "3D print*" OR "three-dimensional print*" OR "direct metal laser sintering" OR DMLS OR "selective laser melting" OR SLM OR "laser melting" OR milling) AND ("atrophic maxilla" OR "atrophic mandible" OR "atrophic jaw" OR "severe jaw atrophy" OR "jaw atrophy" OR "bone deficiency" OR edentulous OR "partial edentulism" OR "fixed partial restoration" OR "oral rehabilitation" OR "dental rehabilitation" OR maxillectomy OR "maxillary defect" OR "mandibular defect" OR "midface defect" OR "maxillofacial reconstruction" OR obturator OR cleft OR agenesis in Title Abstract Keyword	Database inception to 1 May 2026; all Cochrane Library content searched.	0
Manual search	Reference-list screening, citation tracking, grey literature and early online publications	Reference lists of all included articles and relevant reviews, consensus reports, technical notes, and systematic reviews were screened manually. Early online publications and grey literature were retained only when sufficient methodological or clinical information was available.	Performed after electronic database search and deduplication.	3

Abbreviations: AMSJI, additively manufactured subperiosteal jaw implant; CAD/CAM, computer-aided design/computer-aided manufacturing; DMLS, direct metal laser sintering; PSSI, patient-specific subperiosteal implant; SLM, selective laser melting. The search strings above were used as the reproducible electronic strategy and then supplemented by manual screening. The PubMed strategy fulfils PRISMA-ScR Item 8 by providing a complete reproducible strategy for at least one database; the other database strings are reported to maximize transparency.

Supplementary Table S3

Record-to-category mapping of the 56 unique human clinical records with extractable denominators

No.	Reference	Study type	Primary category	Secondary category / overlap	Section/table placement	Counted in 56 unique clinical records	Counted in indication-level assignments	Rationale for assignment
1	Łoginoff et al., 2026	Long-term retrospective study	Full-arch severe atrophy	None	Section 4.1 / Table 1	Yes	Yes	Clinical cohort on DMLS PSSI in severe mandibular atrophy with long-term follow-up.
2	Milad et al., 2026	Case report	Full-arch severe atrophy	Elderly/frail patient	Section 4.1 / Table 1	Yes	Yes	Clinical report of custom 3D-printed PSSI in Cawood VI maxillary atrophy.
3	Łoginoff et al., 2025	Long-term retrospective study	Full-arch severe atrophy	None	Section 4.1 / Table 1	Yes	Yes	Clinical cohort on DMLS PSSI in severely atrophic maxilla with follow-up up to 10 years.
4	Van den Borre et al., 2025	Multicentre retrospective study	Full-arch severe atrophy	Patient-reported outcomes	Section 4.1 / Table 1	Yes	Yes	Clinical cohort on PSSI in severe mandibular atrophy with survival and satisfaction outcomes.
5	Vaira et al., 2024	Multicentre retrospective study	Full-arch severe atrophy	Soft-tissue exposure / success criteria	Section 4.1 / Table 1	Yes	Yes	Multicentre full-arch maxillary cohort with survival, success, exposure, and radiographic outcomes.
6	Gasparini et al., 2024	Retrospective clinical study	Full-arch severe atrophy	Digital CAD-CAM rehabilitation	Section 4.1 / Table 1	Yes	Yes	Clinical study of CAD-CAM subperiosteal implants in atrophic jaws.

No.	Reference	Study type	Primary category	Secondary category / overlap	Section/table placement	Counted in 56 unique clinical records	Counted in indication-level assignments	Rationale for assignment
7	El-Sawy et al., 2024	Clinical case series	Full-arch severe atrophy	PEEK framework	Section 4.1 / Table 1	Yes	Yes	Case series of PEEK subperiosteal framework for full-arch maxillary rehabilitation.
8	Onică et al., 2024	Long-term clinical study	Full-arch severe atrophy	Survival versus success	Section 4.1 / Table 1	Yes	Yes	Long-term clinical study demonstrating discrepancy between implants in situ and successful cases.
9	Van den Borre et al., 2024	Multicentre clinical study	Full-arch severe atrophy	Soft-tissue complications	Section 4.1 / Table 1	Yes	Yes	Multicentre study focused on recession/exposure and mucositis risk factors after maxillary AMSJI.
10	Dimitroulis et al., 2022	Cohort case series	Full-arch severe atrophy	Complications and salvageability	Section 4.1 / Table 1	Yes	Yes	Clinical series reporting primary success, exposure, salvage, and overall success.
11	Nemtoi et al., 2022	Prospective pilot study	Full-arch severe atrophy	Surgical fit / workflow	Section 4.1 / Table 1	Yes	Yes	Prospective clinical pilot study of custom DMLS titanium subperiosteal implants.
12	Van den Borre et al., 2022	Prospective multicentre study	Full-arch severe atrophy	PROMs	Section 4.1 / Table 1	Yes	Yes	Prospective multicentre AMSJI study reporting OHIP-14 and satisfaction outcomes.
13	Van den Borre et al., 2021	Radiographic follow-up study	Full-arch severe atrophy	Bone remodeling	Section 4.1 / Table 1	Yes	Yes	Radiographic study of crestal remodeling and bone response after maxillary AMSJI.
14	Cerea and Dolcini, 2018	Retrospective case series	Full-arch severe atrophy	DMLS titanium framework	Section 4.1 / Table 1	Yes	Yes	Large early clinical series of DMLS titanium subperiosteal implants in atrophic jaws.
15	Mounir et al., 2018	Prospective clinical study	Full-arch severe atrophy	Materials comparison	Section 4.1 / Table 1	Yes	Yes	Clinical comparison of titanium and PEEK patient-specific subperiosteal implants.
16	Vaira et al., 2026	Retrospective case series	Segmental/sectional rehabilitation	Hybrid rehabilitation	Section 4.2 / Table 2	Yes	Yes	Clinical series combining PSSI and conventional endosseous implants in heterogeneous bone availability.
17	Vaira et al., 2025	Cohort study	Segmental/sectional rehabilitation	Posterior maxilla	Section 4.2 / Table 2	Yes	Yes	Clinical cohort on sectional posterior maxillary PSSI in partially dentate patients.
18	Pellegrino et al., 2025	Case series / clinical report with literature review	Segmental/sectional rehabilitation	Preliminary clinical guidelines	Section 4.2 / Table 2	Yes	Yes	Clinical case series of PSSI used across selected partial and complex indications.
19	Darwish et al., 2025	Randomized clinical trial	Segmental/sectional rehabilitation	Milled versus 3D-printed PSSI	Section 4.2 / Table 2	Yes	Yes	Comparative clinical trial in severe mandibular atrophy comparing manufacturing approaches.
20	Cariati et al., 2025	Retrospective case series	Segmental/sectional rehabilitation	Severe maxillary atrophy	Section 4.2 / Table 2	Yes	Yes	Virtual-planned maxillary PSSI case series with three-year clinical outcomes.
21	Vaira et al., 2024	Cohort study	Segmental/sectional rehabilitation	Posterior mandible	Section 4.2 / Table 2	Yes	Yes	Clinical cohort on custom PSSI for severe posterior mandibular atrophy.
22	Vaira et al., 2024	Case report / technical note	Segmental/sectional rehabilitation	Posterior maxillary molar sector	Section 4.2 / Table 2	Yes	Yes	Proof-of-concept sectorial maxillary molar rehabilitation with custom PSSI.
23	Gellrich et al., 2024	Case series	Segmental/sectional rehabilitation	Complex hybrid rehabilitation	Section 4.2 / Table 2	Yes	Yes	Clinical series of complex cases combining PSSI and conventional implants.
24	Nedelcu et al., 2024	Case report	Segmental/sectional rehabilitation	Rescue/salvage	Sections 4.2 and 4.6 / Tables 2 and 5	Yes	Yes, counted in two indication assignments	Posterior mandibular atrophy after previous implant complication; relevant both to sectional rehabilitation and salvage use.

No.	Reference	Study type	Primary category	Secondary category / overlap	Section/table placement	Counted in 56 unique clinical records	Counted in indication-level assignments	Rationale for assignment
25	Fathi et al., 2024	Case report	Segmental/sectional rehabilitation	Mandibular overdenture	Section 4.2 / Table 2	Yes	Yes	Custom mandibular PSSI supporting removable overdenture in severe mandibular atrophy.
26	Arshad et al., 2024	Case report	Segmental/sectional rehabilitation	Legacy/prosthetic rescue	Section 4.2 / Table 2	Yes	Yes	Existing mandibular PSSI used for overdenture support after mandibular fracture management.
27	Krishnaprabhu et al., 2021	Prospective clinical evaluation	Segmental/sectional rehabilitation	Posterior maxilla	Section 4.2 / Table 2	Yes	Yes	Clinical evaluation of hybrid implants in posterior maxillary rehabilitation.
28	Mangano et al., 2020	Case series	Segmental/sectional rehabilitation	Posterior mandible	Section 4.2 / Table 2	Yes	Yes	Case series of custom DMLS titanium PSSI in elderly patients with atrophic posterior mandible.
29	Roy et al., 2025	Case series	Single-tooth rehabilitation	Lateral incisor agenesis	Section 4.3	Yes	Yes	Case series of PSSI for maxillary lateral incisor agenesis in young patients.
30	De Riu et al., 2025	Case report	Congenital/craniofacial rehabilitation	EEC syndrome / cleft-related deformity	Section 4.4 / Table 3	Yes	Yes	Clinical report of PSSI rehabilitation in syndromic cleft-related maxillary deformity.
31	Wirth et al., 2025	Case report	Congenital/craniofacial rehabilitation	Rescue/salvage	Sections 4.4 and 4.6 / Tables 3 and 5	Yes	Yes, counted in two indication assignments	Unrepaired cleft palate with previous failed endosseous and zygomatic implants; relevant to both congenital anatomy and salvage use.
32	Debortoli et al., 2025	Case report / technical note	Congenital/craniofacial rehabilitation	Orthognathic-assisted rehabilitation	Section 4.4 / Table 3	Yes	Yes	PSSI integrated with Le Fort I osteotomy for severe skeletal and prosthetic correction.
33	Ângelo and Ferreira, 2020	Case report	Congenital/craniofacial rehabilitation	Rescue/salvage	Sections 4.4 and 4.6 / Tables 3 and 5	Yes	Yes, counted in two indication assignments	Dental agenesis with previous failed implant rehabilitation; relevant to congenital/hypodontia and rescue indications.
34	Frias et al., 2025/2026	Clinical report	Post-oncologic/post-ablative reconstruction	Obturator retention	Section 4.5 / Table 4	Yes	Yes	Custom maxillary PSSI used for immediate surgical obturator and later implant-retained obturator.
35	De Riu et al., 2025	Case report	Post-oncologic/post-ablative reconstruction	Total maxillectomy + fibula free flap	Section 4.5 / Table 4	Yes	Yes	Primary oncologic reconstruction integrating fibula free flap, PSSI, and delayed prosthetic rehabilitation.
36	De Riu et al., 2025	Retrospective case series	Post-oncologic/post-ablative reconstruction	Primary maxillary reconstruction	Section 4.5 / Table 4	Yes	Yes	Nine-case series of 3D-printed custom PSSI during oncologic maxillary reconstruction.
37	Gellrich et al., 2025	Case series	Post-oncologic/post-ablative reconstruction	Innovative landing zones	Section 4.5 / Table 4	Yes	Yes	Complex maxillary/midfacial defects treated using skull-base, zygomatic, or pterygoid anchorage zones.
38	Segna et al., 2025	Clinical report	Post-oncologic/post-ablative reconstruction	Benign tumor / midface reconstruction	Section 4.5 / Table 4	Yes	Yes	One-step reconstruction after maxillectomy with free fibula flap, custom PSSI, orbital mesh, and immediate prosthesis.
39	John et al., 2025	Case report	Post-oncologic/post-ablative reconstruction	MRONJ-related acquired defect	Section 4.5 / Table 4	Yes	Yes	Patient-specific titanium implant after MRONJ-related jaw resection and rehabilitation.
40	Basavaraju et al., 2024	Case report	Post-oncologic/post-ablative reconstruction	Post-infective acquired defect	Section 4.5 / Table 4	Yes	Yes	Zygoma-supported PSSI after subtotal maxillectomy for post-COVID mucormycosis.
41	Surana et al., 2024	Case report	Post-oncologic/post-ablative reconstruction	Post-infective complication/salvage	Section 4.5 / Table 4	Yes	Yes	Bilateral low-level maxillectomy after mucormycosis with infection and removal of one PSI.
42	De Riu et al., 2023	Case report	Post-oncologic/post-ablative reconstruction	Frail patient / obturator support	Section 4.5 / Table 4	Yes	Yes	Total maxillectomy reconstruction in an elderly patient using custom PSSI and temporal flap.

No.	Reference	Study type	Primary category	Secondary category / overlap	Section/table placement	Counted in 56 unique clinical records	Counted in indication-level assignments	Rationale for assignment
43	Cebrián Carretero et al., 2022	Case series	Post-oncologic/post-ablative reconstruction	Segmental maxillary defects	Section 4.5 / Table 4	Yes	Yes	Customized subperiosteal titanium maxillary implants after oncologic resection.
44	Kondaka et al., 2022	Case report	Post-oncologic/post-ablative reconstruction	Post-infective acquired defect	Section 4.5 / Table 4	Yes	Yes	Zygoma-supported PSI after post-COVID mucormycosis-related maxillary resection.
45	Garrido-Martínez et al., 2022	Case report	Post-oncologic/post-ablative reconstruction	Secondary oncologic rehabilitation	Section 4.5 / Table 4	Yes	Yes	Maxillary SCC treated with resection and subsequent custom subperiosteal implant rehabilitation.
46	Vosselman et al., 2019	Technical note / case report	Post-oncologic/post-ablative reconstruction	Obturator support	Section 4.5 / Table 4	Yes	Yes	Patient-specific subperiosteal zygoma implant for obturator support after subtotal bilateral maxillectomy.
47	Revuelta-Cortés et al., 2026	Case report	Rescue/salvage indications	Post-peri-implantitis severe maxillary atrophy	Section 4.6 / Table 5	Yes	Yes	CAD/CAM PSSI used as rescue strategy after implant failure and peri-implantitis.
48	Cardoso and Grillo, 2025	Case report	Rescue/salvage indications	Failed zygomatic implants	Section 4.6 / Table 5	Yes	Yes	PSSI used after removal of failed or complicated bilateral zygomatic implants.
49	Parras-Hernández et al., 2024	Case report	Rescue/salvage indications	Failed free flap reconstruction / post-infective defect	Section 4.6 / Table 5	Yes	Yes	Personalized subperiosteal implant-supported obturator after failed fibula free flap reconstruction.
50	Korn/Gellrich et al., 2021	Clinical reconstructive report	Cross-cutting clinical evidence	Extended maxillary defects / implant-borne reconstruction	Cross-cutting; not assigned exclusively to Sections 4.1-4.6	Yes	No	Clinical reconstructive evidence focused on implant-borne strategies in extended maxillary defects rather than a single indication category.
51	Machine and Nadjmi, 2025	Clinical report	Cross-cutting clinical evidence	Prosthetic strategy after hemimaxillectomy	Cross-cutting; prosthetic/reconstructive strategy	Yes	No	Clinical report emphasizing prosthetic decision-making, hygiene access, and removable rehabilitation in bulky-flap reconstructive contexts.
52	Diss et al., 2025	Case report	Cross-cutting clinical evidence	Pterygoid anchorage strategy	Cross-cutting; anchorage/design strategy	Yes	No	Clinical report primarily contributing design and anchorage-zone information rather than a new indication category.
53	Pott et al., 2025	Clinical study	Cross-cutting clinical evidence	Coupling elements / design-related complications	Cross-cutting; success criteria and maintenance	Yes	No	Clinical evidence on coupling-element survival, success, spacing, and design-related soft-tissue complications.
54	Vaira et al., 2026	Clinical study	Cross-cutting clinical evidence	Bone apposition / biological response	Cross-cutting; biological response	Yes	No	Clinical evidence focused on bone apposition and biological response around PSSI frameworks rather than a single clinical indication.
55	Van den Borre et al., 2023	Clinical study	Cross-cutting clinical evidence	PROMs and satisfaction	Cross-cutting; patient-reported outcomes	Yes	No	Clinical evidence primarily addressing patient-reported outcomes and satisfaction after AMSJI rehabilitation.
56	Zielinski et al., 2025	Comparative clinical study	Cross-cutting clinical evidence	Zygomatic versus subperiosteal implants	Cross-cutting; comparative graftless rehabilitation	Yes	No	Comparative clinical evidence between zygomatic and subperiosteal implant strategies, used to contextualize graftless alternatives rather than one indication section.

Abbreviations: AMSJI, additively manufactured subperiosteal jaw implant; CBCT, cone-beam computed tomography; CAD/CAM, computer-aided design/computer-aided manufacturing; DMLS, direct metal laser sintering; MRONJ, medication-related osteonecrosis of the jaw; PROMs, patient-reported outcome measures; PSI, patient-specific implant; PSSI, patient-specific subperiosteal implant; SPI, subperiosteal implant.
